# Near‐infrared light-triggered nitric oxide nanocomposites for photodynamic/photothermal complementary therapy against periodontal biofilm in an animal model

**DOI:** 10.7150/thno.83745

**Published:** 2023-04-17

**Authors:** Xiangrong Wu, Manlin Qi, Chengyu Liu, Qijing Yang, Sijia Li, Fangyu Shi, Xiaolin Sun, Lin Wang, Chunyan Li, Biao Dong

**Affiliations:** 1 Department of Prosthodontics, Jilin Provincial Key Laboratory of Tooth Development and Bone Remodeling, School and Hospital of Stomatology, Jilin University, Changchun, 130021, P. R. China.; 2 State Key Laboratory on Integrated Optoelectronics, College of Electronic Science and Engineering, Jilin University, Changchun, 130012, P. R. China.

**Keywords:** Phototherapy, gas therapy, biofilm eradication, nitric oxide, antibacterial, periodontal disease

## Abstract

**Background**: Periodontal disease, an oral disease that initiates with plaque biofilm infection, affects 10% of the global population. Due to the complexity of tooth root anatomy, biofilm resistance and antibiotic resistance, traditional mechanical debridement and antibiotic removal of biofilms are not ideal. Nitric oxide (NO) gas therapy and its multifunctional therapy are effective methods to clear biofilms. However, large and controlled delivery of NO gas molecules is currently a great challenge.

**Methods**: The core-shell structure of Ag_2_S@ZIF-90/Arg/ICG was developed and characterized in detail. The ability of Ag_2_S@ZIF-90/Arg/ICG to produce heat, ROS and NO under 808 nm NIR excitation was detected by an infrared thermal camera, probes and Griess assay. *In vitro* anti-biofilm effects were evaluated by CFU, Dead/Live staining and MTT assays. Hematoxylin-eosin staining, Masson staining and immunofluorescence staining were used to analyze the therapeutic effects *in vivo.*

**Results**: Antibacterial photothermal therapy (aPTT) and antibacterial photodynamic therapy (aPDT) could be excited by 808 nm NIR light, and the produced heat and ROS further triggered the release of NO gas molecules simultaneously. The antibiofilm effect had a 4-log reduction *in vitro*. The produced NO caused biofilm dispersion through the degradation of the c-di-AMP pathway and improved biofilm eradication performance. In addition, Ag_2_S@ZIF-90/Arg/ICG had the best therapeutic effect on periodontitis and NIR II imaging ability* in vivo.*

**Conclusions**: We successfully prepared a novel nanocomposite with NO synergistic aPTT and aPDT. It had an outstanding therapeutic effect in treating deep tissue biofilm infection. This study not only enriches the research on compound therapy with NO gas therapy but also provides a new solution for other biofilm infection diseases.

## Introduction

Periodontal disease, as an inflammatory disease, is the most common cause of missing teeth in adults, affecting approximately 700 million people worldwide 1,2. The key to the treatment of periodontal disease is the removal of dental plaque and colonies of bacterial products on the tooth surface 3, since plaque accumulation is considered to be the initiating factor of the disease. At present, periodontal disease is usually treated with mechanical debridement. Unfortunately, local factors such as deep and narrow periodontal pockets and root anatomical variation may also hinder effective mechanical handling to completely remove the bacteria, compromising the therapeutic effect 4. Additionally, it is also difficult to eradicate biofilms by adjuvant antibiotic therapy with the appearance of antimicrobial resistance and the protective effect from biofilm extracellular matrix 5.

Gas therapy, as a new therapeutic strategy, has attracted extensive investigation interest since it is impossible to cause antimicrobial resistance, and some gas molecules themselves are endogenous signaling molecules 6. Nitric oxide (NO), as one of the most famous therapeutic gas molecules, possesses favorable anti-apoptotic 7 and cancer treatment effects 8. More importantly, NO shows extraordinary broad-spectrum bactericidal performance by peroxidizing lipids, damaging DNA and disrupting the function of proteins 9-11. In addition to direct bactericidal properties, NO is also capable of dispersing bacterial biofilms by interfering with nucleotide signals (c-di-AMP and c-di-GMP) 12 since NO is able to activate phosphodiesterase (PDE, a key enzyme in nucleic acid signal transduction) activity, further reducing the content of nucleic acid signal transduction in biofilms and eventually leading to biofilm disassembly 13-15. Compared with reactive oxygen species (ROS)-based antimicrobial photodynamic therapy (aPDT) and thermal-based antimicrobial photothermal therapy (aPTT), therapeutic gas molecules have greater penetration depth and existence time and have advantages for deep tissue and long-term treatment. To date, a variety of NO donors have already been prepared, such as diazodiol (NONOates) 16 and S-nitrite mercaptan (RSNOs) 17, to realize controllable delivery and exact release of NO. These NO donors can automatically and slowly release NO gas molecules, but byproduct biosafety issues and short half-life will affect their possible application in a physical environment 18. Notably, L-arginine (L-arg) is an amino acid that helps the body build protein, which naturally exists in meat and dairy products and is commonly used in recycling. Because of its special p-guanidine structure in the R group, L-arg becomes a biological NO donor with excellent biosafety, and NO gas molecules can be produced under the catalysis or heat treatment of inducible nitric oxide synthase 19,20.

At present, for this NO gas therapy solution, the challenge is large and controlled delivery of arginine molecules to the lesion site. In addition, gas therapy-based multifunctional therapy methods also need to be developed for more effective treatment effects. To address these issues, in the carrier material system, the introduction of the photothermal effect should be an important way to realize not only the thermally controlled release of gas molecules but also the synergistic effect of photothermal and gas therapy 17. The core-shell thermal release carrier is a classic structure that is composed of a noble metal core with photothermal effects and a shell of mesoporous materials. For example, gold@mSiO_2_ nanocarriers have been used to realize the heat-controlled release of doxorubicin and tumor treatment. Chen et al. 21 and We 22 were the first to develop this kind of thermally controlled release structure, which can support various drug molecules by using silicon channels. This structure has now been further developed, including the carrier design of the photothermal agent@metal organic framework (MOF) structure 23. Note that the organic ligand structure of MOF can achieve specific binding with loaded molecules, and based on this idea, we designed a drug-loading system with silver sulfide (Ag_2_S) nanocrystals as the core and zeolitic imidazole framework-90 (ZIF-90) as the shell for a large load of L-arg. Zeolitic imidazole frameworks (ZIFs), as a member of the metal-organic skeleton family, have many advantages, such as adjustable pore sizes, large specific surface areas and structural diversity 24. ZIF-90, composed of zinc ions (Zn^2+^) and imidazole-2-formaldehyde (2-ICA), is biologically compatible with a variety of cell lines 25. In particular, the aldehyde group of the ZIF-90 organic ligand can bind arginine with a large loading capacity.

**Figure 1:**
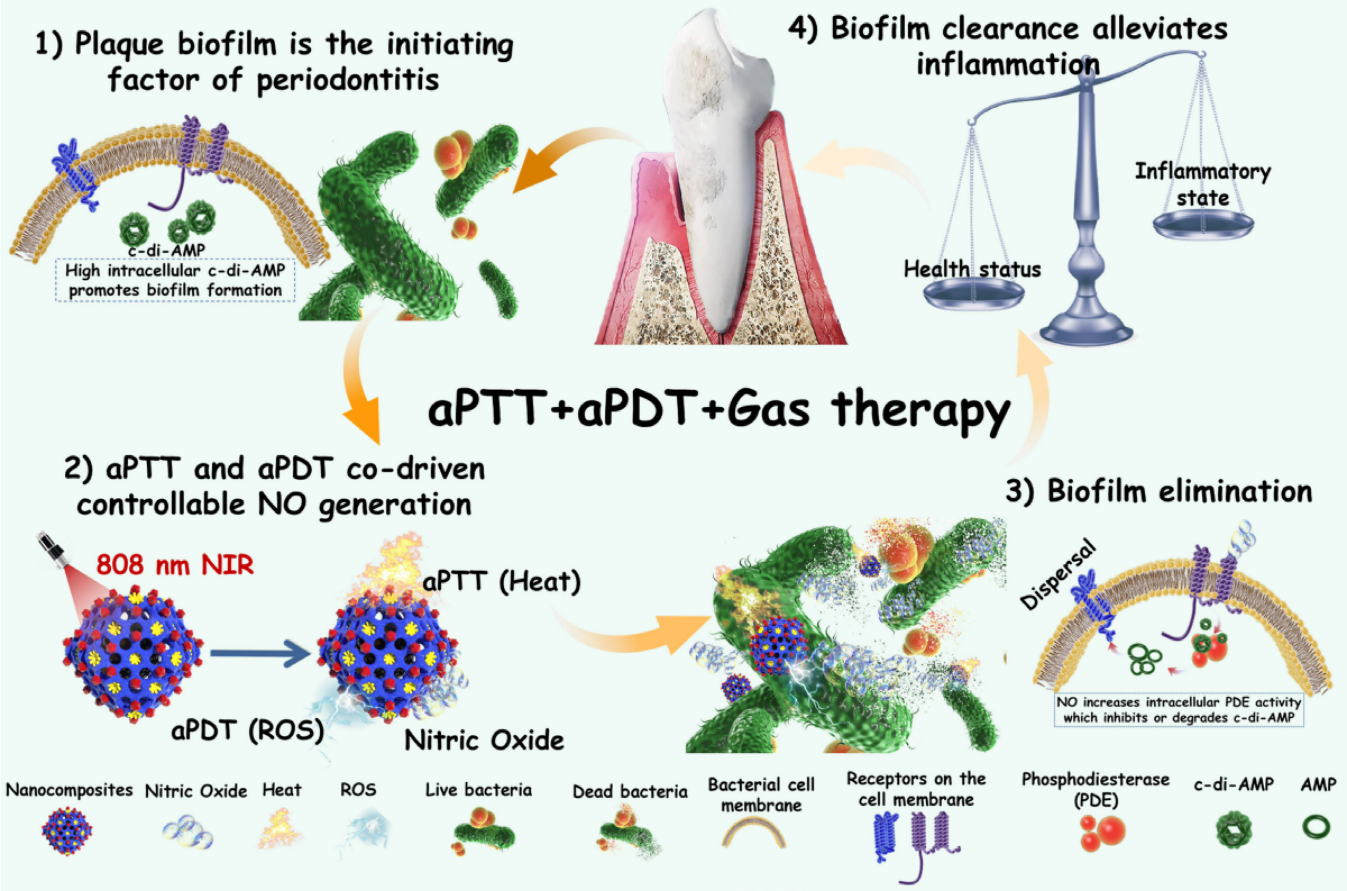
Schematic illustration of the mechanisms involved in the anti-biofilm infection and reduction of periodontal inflammation of Ag_2_S@ZIF-90/Arg/ICG NCs with aPTT/aPDT/NO functions.

Therefore, in this work, we encapsulated photothermal Ag_2_S nanoparticles (NPs) with high photothermal efficiency in ZIF-90 to form core-shell structures (Ag_2_S@ZIF-90). Because the release of NO gas molecules from arginine can also be excited by ROS, aPDT was also involved in this system by introducing indocyanine green (ICG) molecules. Ag_2_S@ZIF-90 was subsequently loaded with L-arg molecules inside the channels by the Schiff Base reaction with ligands of ZIF-90 and modified with ICG on the surface by electrostatic adsorption. Finally, aPTT, aPDT and NO gas therapy were realized by an all-in-one strategy (Ag_2_S@ZIF-90/Arg/ICG), which showed satisfactory biosafety *in vitro* and *in vivo*. Within the Ag_2_S@ZIF-90/Arg/ICG composites, the following features could be expected: 1) Both the photothermal properties of Ag_2_S and the aPDT properties of ICG could be triggered by near infrared (NIR) light with deep penetration; therefore, a single 808 nm NIR could realize multiple functions of aPTT, aPDT and NO gas therapy simultaneously; 2) Because of the special surface structure of ZIF-90, ICG molecules had been modified with good dispersion on the surface, effectively adjusting the photothermal and photodynamic properties as well as the near-infrared fluorescence properties of ICG. 3) The NO-mediated degradation of c-di-AMP pathway through activation of the PDE enzyme can enhance antibacterial and biofilm eradication performance. Such nanoplatforms with diagnostic and therapeutic functions exhibited a great capacity to combat bacterial biofilms, particularly for the treatment of periodontitis (**Figure 1**), and they could potentially be extended to the therapeutics of other biofilm-related infections.

## Materials and methods

### Synthesis of Ag_2_S@ZIF-90/Arg/ICG nanocomposites

Ag_2_S nanoparticles (NPs) were prepared through the hydrothermal process 26. Subsequently, PAA was wrapped on the surface of Ag_2_S NPs to provide carboxy groups for coordination with the Zn^2+^ of ZIF-90. Then, Ag_2_S@ZIF-90 nanocomposites (NCs) were prepared by one-pot self-assembly at 27 °C 27. Finally, the loading of L-arg and ICG was based on previously reported studies with simple modifications 28. The detailed processes are listed in the Supporting Information.

### Characterization of nanocomposites

The microscopic morphology of the nanocomposites was observed by transmission electron microscopy (TEM, H-8100IV, Hitachi, Tokyo, Japan). Biofilms treated with different nanocomposites were observed by scanning electron microscopy (SEM, Carl Zeiss, Oberkochen, Germany). A Shimadzu UV-2550 spectrophotometer (Shimadzu Corporation, Tokyo, Japan) was used to measure the UV‒visible absorption spectrum. The crystal structures of the nanocomposites were recorded by X-ray powder diffraction (XRD, Rigaku, Tokyo, Japan). A zeta potential instrument (Malvern Corporation, England, UK) was used to detect the zeta potential and dynamic light scattering (DLS) of different nanocomposites. The functional groups of various nanocomposites were checked by Fourier transform infrared spectroscopy (FT-IR, Shi-madzu Corporation, Tokyo, Japan). The N_2_ adsorption-desorption isotherms of various nanocomposites were tested by a Micromeritics ASAP 2020 adsorption instrument (Micromeritics Corporation, GA, USA) at -77.3K Elemental analysis was realized by X-ray photoelectron spectrometry (XPS, ESCALab 250, Thermo Fisher Scientific, New York, USA). The photothermal effects of the different nanocomposites were recorded with an infrared thermal imaging camera (FLIR T400, FLIR, Wilsonville, USA). Electron paramagnetic resonance (EPR) spectroscopy was used to detect the types of free radicals by a Bruker EMXplus spectrometer (Bruker Corporation, Massachusetts, USA). The nitric oxide release capacity was measured using nitric oxide kits (Beyotime, Shanghai, China). The production of ROS was characterized with a DPBF probe. 9,10-Anthracenediyl-bis(methylene)dimalonic acid (ABDA) was used to detect singlet oxygen (^1^O_2_). 2,7-Dichlorodihydrofluorescein diacetate (DCFH-DA) was used to capture ROS generated by NCs in biofilms. Detailed procedures are described in the Supporting Information.

### Evaluation of the anti-biofilm activity of NCs stimulated by NIR *in vitro*

*Porphyromonas gingivalis* (*P. gingivalis*, ATCC 33277) and *Fusobacterium nucleatum* (*F. nucleatum*, ATCC 10953) were used for *in vitro* anti-biofilm assays. The mature biofilm culture was similar to that in a previous study 17. Detailed procedures are provided in the Supporting Information. Then, nine groups were established as follows: control, Ag_2_S, Ag_2_S@ZIF-90, Ag_2_S@ZIF-90/ICG, Ag_2_S@ZIF-90/Arg/ICG, Ag_2_S+NIR, Ag_2_S@ZIF-90+NIR, Ag_2_S@ZIF-90/ICG+NIR, and Ag_2_S@ZIF-90/Arg/ICG+NIR. The wells containing mature biofilm were treated with various NCs at a concentration of 0.092 mM. Subsequently, they were irradiated with or without 808 nm NIR (1 W cm^-2^, 5 min) for 3 days. The control group received no treatment. To investigate the efficacy of various nanocomposites on the eradication of mature biofilms, standard plate counting assays, live/dead staining and metabolic activity of biofilms were carried out. The detailed processes are listed in the Supporting Information.

### Gene expression levels of extracellular and intracellular proteins in P. gingivalis

The* P. gingivalis* extracellular proteins peptidylarginine deiminase (*PPAD*)*, HagA, HagB, FimA, RgpA, and RgpB,* genes controlling c-di-AMP synthetase (*pgn 0523*) and catabolic enzymes (*pgn* 1187, *pgn 2003*, *pgn 0521*) were quantitatively detected via RT‒qPCR. Primers for each target gene were constructed by Sangon Biotech (Shanghai, China), as illustrated in **Table S3**. The expression of relevant genes was analyzed for data normalization by the 2^-ΔΔCt^ method. The 16S rRNA gene was set as an internal control for data normalization. The C_t_ value in the control group was used as the calibration value. Each experiment was replicated three times.

### Animal model and therapeutic method

*In vivo* rat periodontal inflammation models were established according to our previous study method 17. Wistar rats (six-week-old, male) were used on the basis of the protocol agreed upon by the Committee for Animal Experimental Ethical Inspection of Jilin University (#SY2022-51), China. Briefly, the rat mandibular anterior teeth were ligated around the cervical margin with (4-0) silk suture. Subsequently, the mixed bacterial suspension (*P. gingivalis* : *F. nucleatum* 1:1) was injected at the site mentioned above. The silk threads were removed after completing the periodontal inflammation model. The Wistar rats were divided into ten groups: Blank control, Negative control, Ag_2_S, Ag_2_S@ZIF-90, Ag_2_S@ZIF-90/ICG, Ag_2_S@ZIF-90/Arg/ICG, Ag_2_S+NIR, Ag_2_S@ZIF-90+NIR, Ag_2_S@ZIF-90/ICG+NIR, and Ag_2_S@ZIF-90/Arg/ICG+NIR. The animal models were treated with various NCs (0.092 mM, 200 μL). Subsequently, 808 nm NIR (1 W cm^-2^, 5 min) was then administered or not at the injection site for 3 days. The negative control group was only treated with 200 μL of sterile saline solution. The blank control group was fed normally without any treatment.

### Antibacterial effect assessment *in vivo*

For* in vivo* antibacterial effect evaluation, the bacterial content of the treated tissue was assessed by standard plate counting at the end of the treatment. In summary, sterile swabs were probed at the site of treatment and then immediately stored in 1 mL of sterile saline solution. Then, bacterial colonies were quantified by the dilution plate method after incubation for 48 h at 37 °C.

### Evaluation of the photothermal effect of Ag_2_S@ZIF-90/Arg/ICG NCs *in vivo*

To evaluate the photothermal effect *in vivo*, Ag_2_S@ZIF-90/Arg/ICG NCs (0.092 mM, 200 µL) were injected into the mandibular incisor, and then rat gingival tissues were irradiated with 808 nm NIR at a power density of 1 W cm^-2^ for 10 min. Thermal images were taken with the imaging camera every 2 minutes.

### Histological evaluation

The gingival tissues at the treatment sites were fixed in 4% paraformaldehyde and embedded in paraffin. H&E staining, Masson's trichrome staining and immunofluorescence staining were used to further estimate the inflammatory status of gingival tissue. Stained sections were observed with a light microscope (Olympus, Tokyo, Japan) at 100× and 400× magnification to count the number of inflammatory cells and to evaluate collagen degradation.

### Real-time PCR assay

Total RNA was extracted from gingival tissue using RNAiso plus (TaKaRa, Otsu, Japan). RT‒qPCR was used to detect the mRNA expression levels of pro-inflammatory (IL-1β) and anti-inflammatory cytokines (Arg-1). All primer sequences used in animal tissue qPCR experiments are listed in **Table S4**. β-actin, as the reference gene, could also be used as an internal control RNA quality test.

### Statistical analysis

The data are expressed as the mean±SD. SPSS 24.0 software (SPSS, Chicago, IL, USA) was used for statistical analyses. One-way analysis of variance (ANOVA) was performed with Tukey's HSD posttest. When p<0.05, the difference in values was considered significant.

## Results and Discussion

### Construction and characterization of Ag_2_S@ZIF-90/Arg/ICG NCs

**Figure 2:**
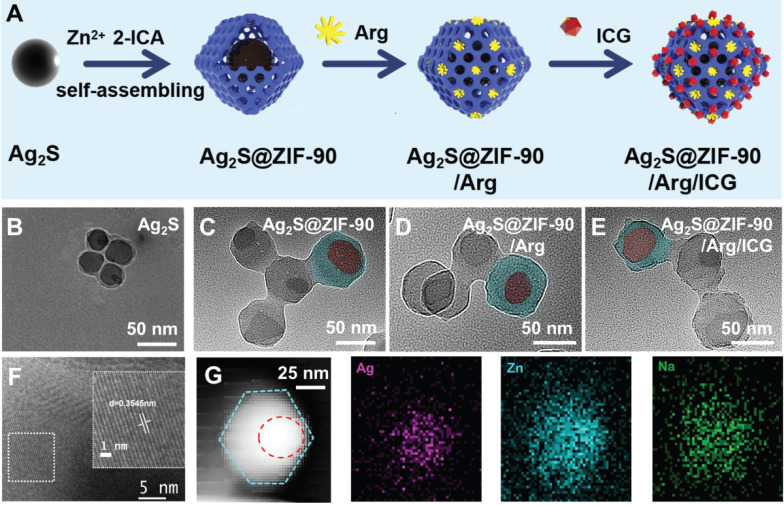
** Characterization of various NCs.** (A) The synthesis process of Ag_2_S@ZIF-90/Arg/ICG. (B-E) Representative TEM images of (B) Ag_2_S, (C) Ag_2_S@ZIF-90, (D) Ag_2_S@ZIF-90/Arg, and (E) Ag_2_S@ZIF-90/Arg/ICG (the red rendering represents Ag_2_S, and the blue rendering represents ZIF-90). (F) HR-TEM image of Ag_2_S. (G) Elemental mappings of Ag, Zn and Na of Ag_2_S@ZIF-90/Arg/ICG.

The synthetic procedures of Ag_2_S@ZIF-90/Arg/ICG NCs are illustrated in **Figure 2A**. Ag_2_S NPs were synthesized via a one-pot hydrothermal method 26. PAA was used as a surfactant to prevent Ag_2_S NPs from aggregation, and it could also provide -COOH groups for further connection to Zn^2+^, making the ZIF-90 shell grow on Ag_2_S NPs 27. In the synthesis process, L-arg molecules were loaded inside the pores of the ZIF-90 shell layer via the Schiff base reaction 27. Finally, ICG was loaded into ZIF-90 via electrostatic attraction with high loading efficiency because ICG molecules can form π-π stacking interactions 28. TEM images of nanocomposites at different stages are shown in **Figure 2B-2E**. Ag_2_S NPs were uniformly distributed in spheres with a diameter of approximately 35 nm. Dynamic light scattering (DLS) was used to test different NC size changes (**Figure S1**). The particle sizes of different NCs obtained by DLS and TEM are summarized in **Table S1**. It is well known that the size of nanoparticles measured by DLS is usually larger than that observed by TEM 29. This is because DLS measures the radius of hydrated particles, while the particle size displayed by TEM is the radius of dried nanoparticles. The HR-TEM image in **Figure 2F** shows that the Ag_2_S NPs were equipped with a monoclinic crystal system possessing a favorable crystallographic structure and a lattice stripe of 0.3545 nm. After assembly onto PAA-coated Ag_2_S NPs, the structure of the Ag_2_S@ZIF-90 core-shell could be observed. There were no obvious changes that could be seen after step-by-step loading of L-arg and ICG molecules in ZIF-90. The elemental composition (Ag, Zn and Na) of Ag_2_S@ZIF-90/Arg/ICG NCs provided by element mapping in** Figure 2G** not only confirmed the core-shell structure of Ag_2_S@ZIF-90 but also showed the successful loading of ICG molecules on ZIF-90.

**Figure 3:**
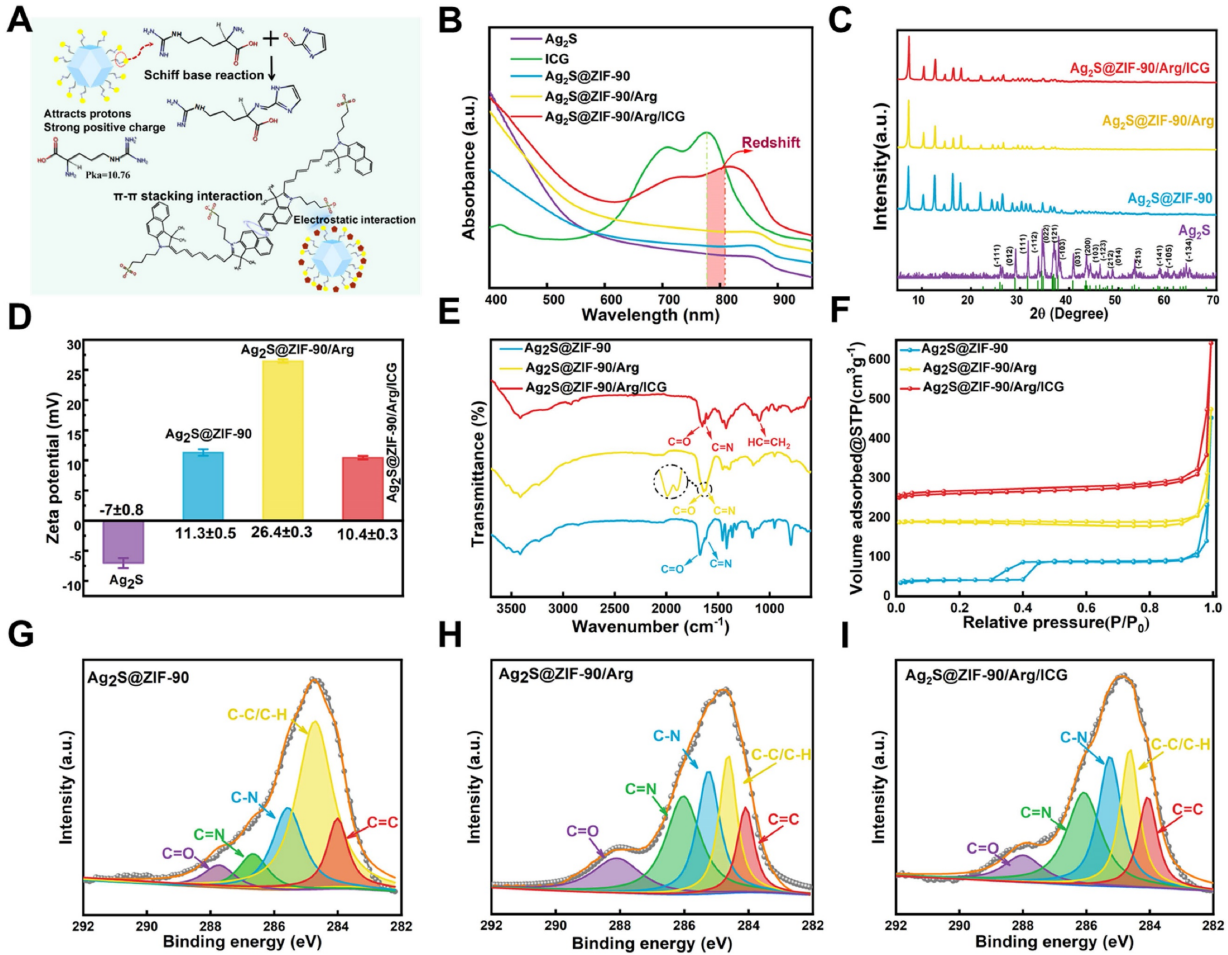
** Characterization of various NCs.** (A) Schematic representation of L-arg and ICG loaded into Ag_2_S@ZIF-90. (B) UV‒vis absorption spectra of various NCs. (C) XRD analysis of various NCs. (D) Zeta potential of various NCs. (E) FT-IR spectra of various NCs. (F) N_2_ adsorption-desorption isotherm of various NCs. (G-I) High-resolution XPS spectra of C 1 s of various NCs.

The modification method of L-arg and ICG in the nanocomposites is shown in** Figure 3A**. In short, the -NH_2_ of L-arg chemically bonded to the -CHO of ZIF-90 to generate imine (C=N) and substituted H_2_O molecules. ICG is an aromatic compound, and the aromatic rings between ICG have noncovalent bond interactions (π-π stacking) as important as hydrogen bonds. The UV‒vis spectra of ICG, Ag_2_S NPs, Ag_2_S@ZIF-90 NCs, Ag_2_S@ZIF-90/Arg NCs, and Ag_2_S@ZIF-90/Arg/ICG NCs are shown in **Figure 3B**. The characteristic peak of pure ICG appeared between 710 and 810 nm. For Ag_2_S@ZIF-90/Arg/ICG NCs, the absorption peak of ICG could also be observed, but a redshift could be found when compared to the pure ICG absorption peak, which should be caused by the π-π stacking of ICG. All diffraction peaks of Ag_2_S NPs could be attributed to the monoclinic α-Ag_2_S standard PDF card (JCPDS file No.14-0072) 26,30, and other impurity peaks were not detected in the XRD patterns (**Figure 3C**). As ZIF-90 grew on the surface of Ag_2_S NPs and formed a core-shell structure Ag_2_S@ZIF-90, only the diffraction peaks of ZIF-90 could be observed because of the encapsulation of ZIF-90 27, and this result also proved that the Ag_2_S NPs were thoroughly and completely wrapped by the MOF structure. The characteristic peaks of Ag_2_S@ZIF-90/Arg NCs and Ag_2_S@ZIF-90/Arg/ICG NCs did not change compared with that of Ag_2_S@ZIF-90 NCs, meaning that the skeleton construction of ZIF-90 was not destroyed by the modification of L-arg and ICG.

Additionally, the zeta potential data in **Figure 3D** further established the successful encapsulation of the Ag_2_S@ZIF-90/Arg/ICG NCs. The surface of Ag_2_S NPs showed a negative charge (-7.0 ± 0.8 mV). After modification by PAA, the -COOH provided by PAA increased the negative charge on its surface, which can attract more Zn^2+^ to coordinate with 2-ICA to form ZIF-90 on the surface. Therefore, the surface charge of Ag_2_S@ZIF-90 NCs was positive. When L-arg molecules were loaded into ZIF-90 pores, since the isoelectric point of L-arg (Pka=10.76) was higher than that of the aqueous solution, the amino group was protonated (-NH^3+^), which increased the positive charge on the ZIF-90 surface. ICG had a strong negative charge property and could be attracted to the positively charged surface of Ag_2_S@ZIF-90/Arg NCs by static electricity. Correspondingly, the surface charge of Ag_2_S@ZIF-90/Arg/ICG NCs decreased. The FT-IR spectra of various NCs is shown in **Figure 3E**. There was a peak at 1677 cm^-1^ resulting from the C=O stretching vibration of the aldehyde group on the Ag_2_S@ZIF-90 NCs imidazole ring. Compared to Ag_2_S@ZIF-90 NCs, the C=O stretching vibration moved toward the lower wavenumber due to the bimolecular association in the arginine carboxyl molecule. Moreover, an increase in the C=N double bond ratio was observed 31. We speculated that this was due to the Schiff base reaction between -NH_2_ of L-arg and -CHO of 2-ICA to produce the carbon-nitrogen double bond. When this occurred, the corresponding absorption of the aldehyde group also decreased, as seen in the spectrum. Moreover, the emergence of a unique peak at 1097 cm^-1^ belonging to the vinyl stretch of ICG indicated the successful loading of ICG 28.

Since N_2_ adsorption/desorption is an effective characterization method for porous materials, it is used to investigate the specific surface area and pore changes of ZIF-90 to further confirm the specific positions of L-arg and ICG loaded in ZIF-90. N_2_ adsorption/desorption experiments were performed to ascertain the properties of various NCs at 77.3 K 32. Specimens were degassed at 120 °C for two hours before formal testing. There was obvious hysteresis in the desorption curve of Ag_2_S@ZIF-90 NCs (**Figure 3F**), indicating that this curve was a type IV curve, which signified those abundant mesoporous structures existed in the Ag_2_S@ZIF-90 NCs. Nevertheless, the desorption curves and properties of the Ag_2_S@ZIF-90/Arg NCs and Ag_2_S@ZIF-90/Arg/ICG NCs showed type III curves characteristic of microporous materials. The BET and Langmuir surface areas of Ag_2_S@ZIF-90 NCs, Ag_2_S@ZIF-90/Arg NCs and Ag_2_S@ZIF-90/Arg/ICG NCs were (823.753 m²/g, 203.933 m²/g and 33.561 m²/g) and (1036.510 m²/g, 271.587 m²/g and 41.037 m²/g), respectively (**Table S2**). The significant reduction in pore volume compared to Ag_2_S@ZIF-90 NC crystals was due to the narrowing of pore volume by L-arg and ICG modification.

Furthermore, the NCs were measured by X-ray photoelectron spectroscopy (XPS) (**Figure S2**). As illustrated in** Figure 3G-3I**, the C 1s spectra of Ag_2_S@ZIF-90 NCs depicted that the characteristic peaks emerged at the following locations: C-H/C-C (284.7 eV), C-N (285.6 eV), C=C (284 eV), C-O (286.3 eV), C=N (286.6 eV) and C=O (287.7 eV) 27. The C=O of Ag_2_S@ZIF-90 was provided by 2-ICA, and the carboxyl group of L-arg in Ag_2_S@ZIF-90/Arg NCs led to an increase in the C=O ratio (3.37% *vs.* 11.36%) (**Figure S3**). However, the C=O ratio decreased after further modification by ICG. The C=N of Ag_2_S@ZIF-90 NCs was also provided by 2-ICA. The loading of L-arg onto the Ag_2_S@ZIF-90 NCs surface resulted in an increase in the C=N ratio of Ag_2_S@ZIF-90/Arg NCs (6.13% *vs.* 27.18%), as shown in **Figure S3**, and the C=N ratio was basically unchanged after further modification in Ag_2_S@ZIF-90/Arg/ICG NCs. The original C-N of Ag_2_S@ZIF-90 NCs was provided by 2-ICA. Thereafter, the increase in the C-N proportion of Ag_2_S@ZIF-90/Arg/ICG NCs was ascribed to the loading of L-arg. Likewise, as depicted in the S 2p spectra (**Figure S4**) obtained from Ag_2_S@ZIF-90/Arg/ICG NCs, the S element was contributed by the sulfonic acid group (-SO_3_^2-^, 168.5 eV) of ICG. The ninhydrin method was used to determine the drug loading and encapsulation efficiency of L-arg as 21.46% and 74.14%, respectively (**Figure S5**). The drug loading and encapsulation efficiency of ICG loaded on Ag_2_S@ZIF-90/Arg/ICG NCs were 5.31% and 81.21%, respectively (**Figure S6**). Taken together, the above characterization proved the successful synthesis of Ag_2_S@ZIF-90/Arg/ICG NCs.

### *In vitro* thermal, ROS and NO generation behavior of Ag_2_S@ZIF-90/Arg/ICG

**Figure 4:**
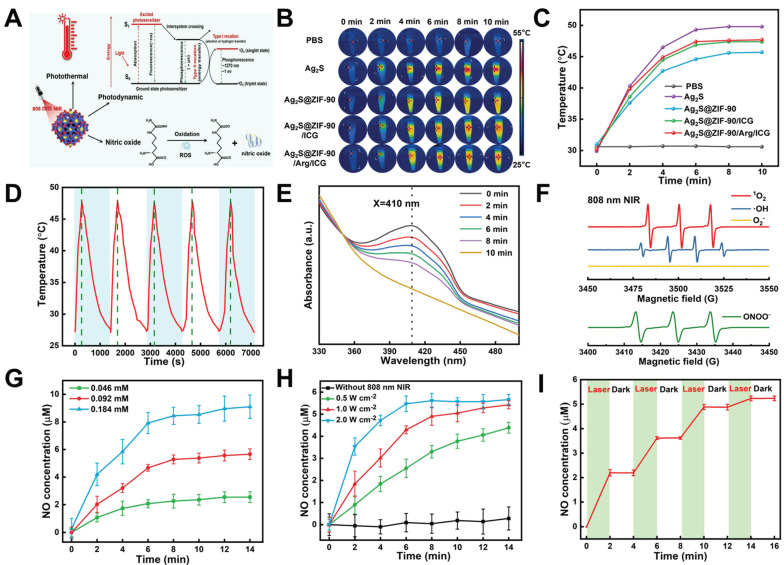
** The ability of Ag_2_S@ZIF-90/Arg/ICG NCs to produce heat, ROS and NO under NIR excitation.** (A) Schematic illustration mechanism of Ag_2_S@ZIF-90/Arg/ICG NCs for the generation of thermal, ROS and NO molecules. (B) Infrared thermal photos of various NCs irradiated with 1 W cm^-2^ power density NIR light for 10 min and (C) Relevant temperature curve record plot. (D) Photothermal stability of Ag_2_S@ZIF-90/Arg/ICG NCs (0.092 mM) for 5 cycles under NIR laser irradiation (1 W cm^-2^). (E) The consumption of DPBF reacting with singlet oxygen generated by Ag_2_S@ZIF-90/Arg/ICG NCs (0.092 mM) under NIR laser irradiation (1 W cm^-2^). (F) EPR spectrum of reactive radicals ^1^O_2_, ·OH, O_2_^-^ and ONOO^-^ with 808 nm NIR irradiation. (G) The amount of NO release within 15 min irradiation (1 W cm^-2^) on Ag_2_S@ZIF-90/Arg/ICG NCs at different concentrations (0.046 mM, 0.092 mM, and 0.184 mM). (H) The cumulative NO release of the same concentration of Ag_2_S@ZIF-90/Arg/ICG (0.092 mM) NCs at different power densities (0.5 W cm^-2^, 1 W cm^-2^, 2 W cm^-2^). (I) NO release behavior controlled by an 808 nm NIR (1 W cm^-2^, 15 min) switch.

As shown in **Figure 4A**, the aPTT, aPDT and NO generation mechanism of Ag_2_S@ZIF-90/Arg/ICG NCs was illustrated. Ag_2_S NPs were previously reported as an important photothermal conversion agent. ICG is one of the few photosensitizers authorized by the US Food and Drug Administration for clinical use 33. In UV‒vis spectra, Ag_2_S@ZIF-90/Arg/ICG NCs had a wide absorption spectrum of 650-850 nm, demonstrating that they could be excited by light with much deeper tissue penetration. Therefore, after successfully constructing Ag_2_S NPs, Ag_2_S@ZIF-90 NCs Ag_2_S@ZIF-90/ICG NCs, and Ag_2_S@ZIF-90/Arg/ICG NCs, they were triggered by an 808 nm NIR laser, and photos were taken by an infrared thermal imaging camera. All NCs at different stages had the same molar concentration, and the concentration of Ag_2_S@ZIF-90/Arg/ICG was 0.092 mM under 808 nm NIR excitation. The power density of the 808 nm NIR laser was 1 W cm^-2^, and the samples were irradiated for 10 min under the same environment. Photothermal photographs and corresponding temperature records are shown in **Figure 4B-4C.** The results revealed that all the NCs had a photothermal effect superior to that of PBS. Within 10 minutes of irradiation, the temperature increased with the passage of irradiation time, especially for the pure Ag_2_S NPs. In this experiment, the photothermal conversion efficiency of Ag_2_S NPs and Ag_2_S@ZIF-90/Arg/ICG NCs reached 47.6% and 36.2%, respectively (**Figure S7-S8**). As depicted in **Figure S9A-S9D**, the temperature variation, as well as the corresponding temperature change curves of Ag_2_S@ZIF-90/Arg/ICG NCs, were recorded at different concentrations and laser powers, and the results indicated the photothermal feature of concentration dependence and laser power dependence. When Ag_2_S NPs were coated with ZIF-90, the photothermal effect was not as ideal as that of pure Ag_2_S NPs. Interestingly, the photothermal effect of Ag_2_S@ZIF-90/Arg/ICG NCs was improved again, which was related to the fact that ICG itself can exert photothermal effects. As a supplement, we also measured the photothermal effect of ICG and found that the total increase in ICG temperature was only 8 °C after 10 min (**Figure S9E-S9F**), which was associated with the light energy consumption caused by the photodynamic generation of ICG under 808 nm irradiation. Moreover, as shown in **Figure 4D**, the photothermal efficacy of Ag_2_S@ZIF-90/Arg/ICG NCs was almost unchanged after 5 irradiation cycles under the same conditions, suggesting that the Ag_2_S@ZIF-90/Arg/ICG NCs had favorable photothermal stability.

Apart from having perfect aPTT performance, Ag_2_S@ZIF-90/Arg/ICG NCs also revealed excellent aPDT performance. The DPBF probe was used to detect the ^1^O_2_ generation of Ag_2_S@ZIF-90/Arg/ICG NCs under 808 nm near-infrared light, as shown in **Figure 4E** and **Figure S10A**. In general, the absorption peak of DPBF is 410 nm, and its content would be irreversibly consumed with the production of singlet oxygen ^1^O_2_, resulting in a reduction in the absorption value at 410 nm. ROS quantitative analysis found that the concentration of ROS produced by 0.092 mM Ag_2_S@ZIF-90/Arg/ICG NCs was approximately 763.95 nM (**Figure S10B**). In addition, ABDA was used as a specific probe for singlet oxygen (^1^O_2_) to investigate the ability of free ICG and loaded ICG to produce ^1^O_2_ (**Figure S11**). Surprisingly, the loaded ICG was better at producing ^1^O_2_ than the free ICG. According to the law of conservation of energy, when Ag_2_S NPs in Ag_2_S@ZIF-90/Arg/ICG NCs absorbed light energy and generated heat rapidly, resulting in a rise in ambient temperature, ICG distributed on the surface was more inclined to divert light energy to produce singlet oxygen 34,35. Furthermore, electron paramagnetic resonance (EPR) was used to determine the type of free radicals produced by Ag_2_S@ZIF-90/Arg/ICG NCs. ^1^O_2_, ·OH, and ONOO^-^ signals could be detected, but O_2_^-^ was not detected (**Figure 4F and Figure S12**). In the biological field, the production of L-arg to nitric oxide by ROS is a mature technology 36. Irradiating Ag_2_S@ZIF-90/Arg/ICG NCs with 808 nm NIR light provided an intelligent and controllable method to achieve NO release in response to ROS. The Griess test was used to characterize NO gas production. Assisted by the standard curve in **Figure S13A**, NO gas production was quantified. For NIR-triggered Ag_2_S@ZIF-90/Arg/ICG NCs, the production of NO showed concentration dependence and power density dependence, as shown in **Figure 4G-4H**. As shown in** Figure 4I**, when 808 nm near-infrared light was turned on, NO was generated within 2 minutes, but when excitation light was turned off, NO was not generated, indicating that the NO gas of Ag_2_S@ZIF-90/Arg/ICG NCs can be generated by a laser trigger. In **Figure S13B**, we found that except for Ag_2_S@ZIF-90/Arg/ICG NCs, NO production of NCs at different stages was not detected under 808 nm NIR light. When the 808 nm NIR laser was absent, neither ROS nor NO was produced, as shown in **Figure S14**. As expected, the synthesized Ag_2_S@ZIF-90/Arg/ICG NCs resulted in a cascade of photothermal effects and ROS-dependent NO gas release under 808 nm NIR irradiation. In addition, it is of significance to evaluate the stability of Ag_2_S@ZIF-90/Arg/ICG NCs for their further application. As shown in **Figure S15A-15B**, UV‒vis spectra and zeta potential proved that Ag_2_S@ZIF-90/Arg/ICG NCs were basically stable in PBS solution over time. The pH value of sulci fluid is critical to the structure and function of periodontal tissue 17. A rise in pH from 6.90 to 8.66 usually occurs when inflammation increases 17. The onset of periodontitis is also associated with an increase in the pH of gingival crevicular fluid to approximately 8.5 due to protein degradation by bacteria 17. Therefore, as shown in **Figure S16A-16B**, the stability of Ag_2_S@ZIF-90/Arg/ICG NCs under different pH values (pH=5.5, 7.0, and 8.5) was tested to simulate physiological and pathological stability. The Ag_2_S@ZIF-90/Arg/ICG NCs attracted negative charges in the alkaline environment, so a slight decrease in the zeta potential value was observed. In contrast, Ag_2_S@ZIF-90/Arg/ICG NCs could obtain positive charges in the acidic environment, and the corresponding zeta potential value increased. Although changes in pH value caused changes in their zeta potential values, Ag_2_S@ZIF-90/Arg/ICG NCs remained positively charged. The fluorescence intensity of Ag_2_S@ZIF-90/Arg/ICG NCs in the NIR-II region hardly changed at different times and pH conditions (**Figure S15C and S16C**). Moreover, as shown in **Figure S15D and S16D**, the Tyndall effect of the Ag_2_S@ZIF-90/Arg/ICG NCs colloidal solution was unaffected at different times and pH values. In summary, the excellent colloidal stability of Ag_2_S@ZIF-90/Arg/ICG NCs was confirmed by UV‒vis, zeta potential, NIR-II imaging and the Tyndall effect. Notably, the ability of Ag_2_S@ZIF-90/Arg/ICG NCs to produce heat, ROS and NO remained unchanged at different pH values (**Figure S17**), demonstrating the uncompromised capacity of nanomedicine in different microenvironments.

### Antibacterial effects of various NCs against mature biofilm

**Figure 5:**
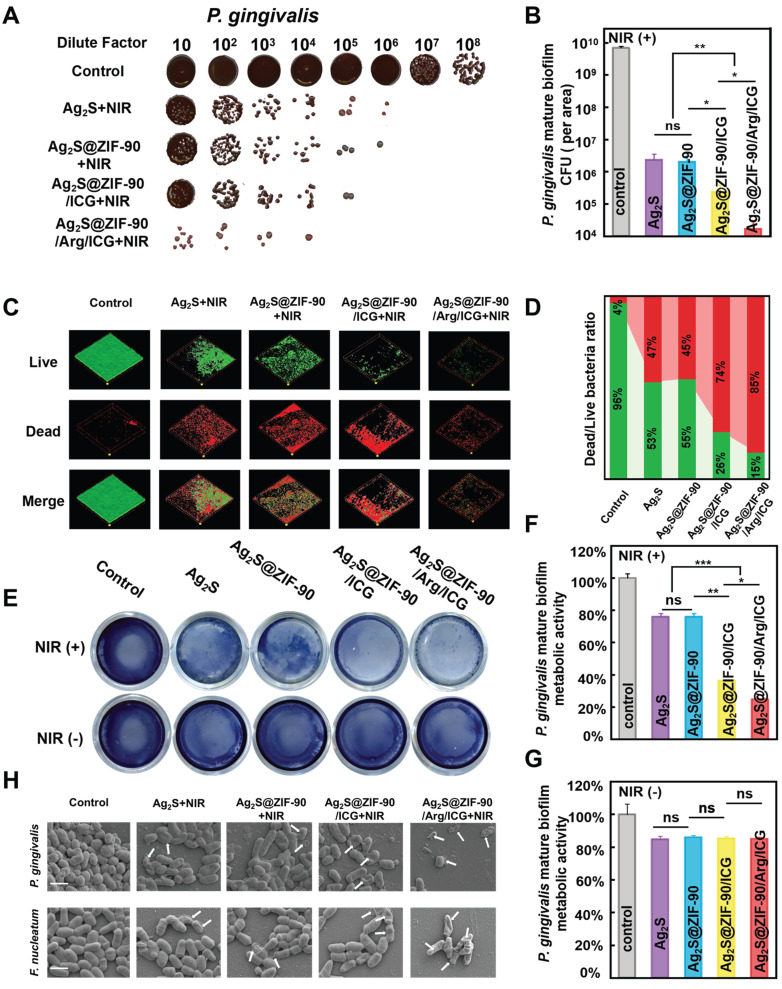
** The capacity of various nanocomposites to resist *P. gingivalis* biofilms under NIR irradiation.** (A) Images of *P. gingivalis* clones and (B) corresponding statistical data of colonies of *P. gingivalis*. (C) Dead and live *P. gingivalis* staining in mature biofilms with various nanocomposite treatments (red fluorescence represents dead bacteria; green fluorescence represents live bacteria) and (D) the corresponding proportion of live and dead bacteria in *P. gingivalis* biofilms. (E) Photographs and test results of the biofilm metabolic activity of *P. gingivalis* by MTT assay and (F-G) corresponding statistical data of biofilm metabolic activity. (H) SEM images of* P. gingivalis* and *F. nucleatum* biofilms. Damage and degeneration of the cell membrane are marked with white arrows. Scale bar: 1 μm (n = 3, *p < 0.05, **p < 0.01, ***p < 0.001, ns, not significant).

Inspired by the outstanding results above, the *in vitro* anti-biofilm properties of Ag_2_S@ZIF-90/Arg/ICG NCs were further evaluated upon 808 nm NIR irradiation against *P. gingivalis* and *F. nucleatum*, two typical bacteria in the red complex and orange complex of periodontitis pathogens in periodontal disease 37,38. The concentrations of anti-free *P. gingivalis* and *F. nucleatum in vitro* were 0.023 mM and 0.046 mM by minimum inhibitory concentration (MIC) and minimum bactericidal concentration (MBC), respectively, as shown in **Figure S18A-S18B** 39. Because the *in vitro* safe concentration of Ag_2_S@ZIF-90/Arg/ICG NCs was 0.092 mM, 0.092 mM was selected as the anti-biofilm concentration in the following experiments. Time-killing curves of 0.092 mM Ag_2_S@ZIF-90/Arg/ICG NCs against *P. gingivalis* and *F. nucleatum* are shown in **Figure S18C-S18D**. Under 808 nm NIR conditions, Ag_2_S@ZIF-90/Arg/ICG NCs killed bacteria in a time-dependent manner, and two strains of bacteria had killing times of 6 h. In addition, **Figure S19A-S19B** shows that the green fluorescence emitted by 2,7-dichlorofluorescin diacetate (DCFH-DA) capturing ROS could be observed in the Ag_2_S@ZIF-90/Arg/ICG NCs group compared to the control group. After quantifying ROS produced by Ag_2_S@ZIF-90/Arg/ICG NCs in the biofilm (**Table S5**), it was found that ROS production decreased compared with previous quantitative testing (**Figure S10B**). This was because biofilms were dense and oxygen-deficient, which could weaken the PDT effect and ultimately lead to lower ROS production. In addition, NO generated by Ag_2_S@ZIF-90/Arg/ICG NCs in biofilms could also be detected, as shown in **Figure S19C-S19D**. **Figure 5A** depicts significantly reduced colony formation counts of *P. gingivalis* for all experimental groups under 808 nm NIR irradiation compared with the control group. In particular, Ag_2_S@ZIF-90/Arg/ICG+NIR possessed the strongest antibiofilm efficiency among all groups. As plotted in **Figure 5B**, the anti-biofilm effect reached a 2-log reduction in *P. gingivalis* biofilm by treatment with Ag_2_S+NIRmediated aPTT and Ag_2_S@ZIF-90+NIRmediated aPTT. However, CFU of *P. gingivalis* biofilm via treatment of Ag_2_S@ZIF-90/ICG+NIRmediated aPTT/aPDT had a 3-log reduction. Interestingly, with the assistance of NO therapy, the anti-biofilm capacity was further enhanced, with a 4-log reduction of *P. gingivalis* biofilm in the Ag_2_S@ZIF-90/Arg/ICG+NIR groups (aPTT/aPDT/NO) upon NIR irradiation in contrast to the control group. In general, once bacteria form biofilms, they produce massive polymers such as polysaccharides and lipids to wrap the bacteria, thus forming a hard physical barrier. Biofilms can prevent external stimuli from killing internal bacteria, which increases the difficulty of biofilm removal. Antimicrobial photothermal therapy alone requires a higher dose of photothermal agents and much greater power to eradicate biofilms, which may burn normal tissues 40. Previous research has confirmed that ROS can disrupt the extracellular matrix and degrade biofilm components, leading to biofilm dispersion 41. For example, ROS can react with ribonucleic acids, leading to the disruption of DNA bonds and the collapse of the extracellular DNA double helix structure 41. Although highly effective, one critical defect of aPDT is that the penetration radius of ROS is exceedingly short, approximately tens to hundreds of nanometers, and the lifespan is 3.5 µs 42, inevitably damaging the antibacterial property against pathogenic biofilms. Remarkably, NO molecules, as free radicals, could not only weaken the biofilm antioxidant system but also degrade c-di-AMP 15. Moreover, the dispersed biofilm did not become denser, which was conducive to improving the permeability of ROS and giving it better performance. In addition, owing to the intrinsic superiority of the long lifespan of NO (approximately 5 s) and far penetration radius (40-200 µm) 42, which could exactly compensate for the deficiency of the bactericidal area of ROS, NO molecules have a crucial advantage in improving the anti-biofilm effect. At the same time, as reported, NO could react with ROS to generate more lethal peroxynitrite (ONOO^-^) molecules, which effectively destroyed the bacterial cell membrane 28. Therefore, NO molecules served as favorable assistance for the treatment of aPTT+aPDT in anti-biofilm application with enhanced CFU reduction. Notably, when no 808 nm NIR light was involved, the CFU results (**Figure S20A-S20B**) were reduced by only 1 log for all experimental groups, which was similar to that of the reported Ag_2_S NPs 43 and Zn^2+^-containing antimicrobial MOFs 31.

In addition, live/dead staining **(Figure 5C**-**5D** and **Figure S20C-S20D)** was used to continue to verify the antibiofilm effect. Identical to the CFU results, the proportion of live bacteria (green staining) in all groups without 808 nm NIR irradiation was almost identical to that of the control, and moreover, the biofilm area did not decrease. In sharp contrast, dead bacteria accounted for the highest proportion in Ag_2_S@ZIF-90/Arg/ICG+NIR. Compared with the residual biofilm area of the aPTT groups (Ag_2_S+NIR and Ag_2_S@ZIF-90+NIR) and aPTT/aPDT group (Ag_2_S@ZIF-90/ICG+NIR), the dead bacteria in the Ag_2_S@ZIF-90/Arg/ICG+NIR group accounted for a large proportion (85%), and the area of the remaining biofilm was dramatically reduced, which could be attributed to the long life and large diffusion radius of NO. The metabolic activities of the single species biofilm (**Figure 5E-5G**) of *P. gingivalis* were similar to the CFU results and live/dead staining. Meanwhile, the anti-biofilm effect of all groups against *F. nucleatum* was also investigated by CFU counts, live/dead staining, and metabolic activities (**Figure S21**). A resemblance was detected in the antibacterial capacity against *F. nucleatum* biofilm, probably due to the similar cell structure (gram-negative bacteria) and respiratory system (strict anaerobes) between *F. nucleatum* and *P. gingivalis.* On this basis, the antibiofilm effects of various NCs under 808 nm NIR were qualitatively evaluated by SEM. As shown in **Figure 5H**, the Ag_2_S@ZIF-90/Arg/ICG+NIR group with aPTT, aPDT and NO therapy had a minimum biofilm area compared with the control group and other experimental groups. The remaining bacteria had either the bacterial cell membrane destroyed or the bacterial skeleton collapsed. However, the bacterial structure in the other experimental groups remained nearly intact, which was consistent with the results of the CFU and MTT experiments. Hence, the designed Ag_2_S@ZIF-90/Arg/ICG NCs triggered by 808 nm NIR light possessed an excellent antibacterial effect with NO-complemented aPTT/aPDT treatment on the dissipation of biofilms, thus providing new potential for removing stubborn periodontal plaque biofilms.

### The biofilm invasiveness changes and underlying mechanism of Ag_2_S@ZIF-90/Arg/ICG NCs

**Figure 6:**
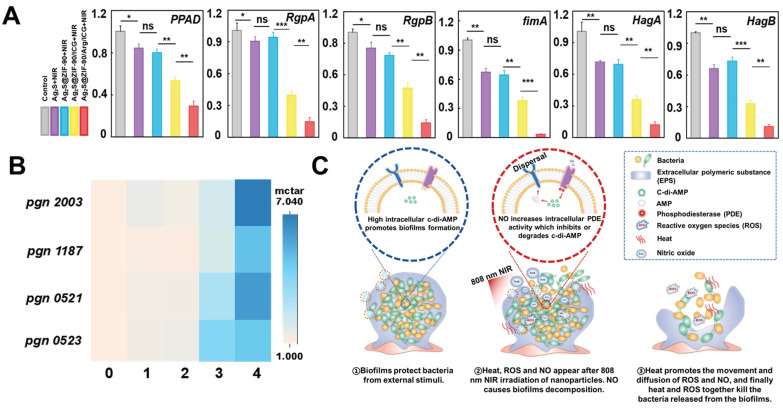
** The effect of various NCs on the gene expression of *P. gingivalis*-related virulence factors and the expression of c-di-AMP pathway synthetase and catabolic enzymes under laser irradiation.** (A) The relative mRNA expression level of pathogenic genes associated with *P. gingivalis* biofilms treated by various NCs with 808 nm NIR excitation. (B) Heatmap of the expression of the c-di-AMP synthase gene *pgn 0523* and hydrolase *pgn 0521*, *pgn 1187* and *pgn 2003* in different nanocomposite experimental groups under 808 nm laser irradiation (different numbers represent different groups. 0 means “control”, 1 means “Ag_2_S+NIR”, 2 means “Ag_2_S@ZIF-90+NIR”, 3 means “Ag_2_S@ZIF-90/ICG+NIR” and 4 means “Ag_2_S@ZIF-90/Arg/ICG+NIR”). (C) Diagram of thermal and ROS bactericidal mechanisms after NO-guided biofilm dispersion. (n = 3, *p < 0.05, **p < 0.01, ***p < 0.001, ns, not significant.)

The* P. gingivalis* LPS profile and virulence potential change were concomitant with c-di-AMP signaling alterations in the infection model 44. Without c-di-AMP catabolic enzyme, *P. gingivalis* showed higher levels of endotoxin immune reactivity, as well as virulence and toxicity 44. In this study, *P. gingivalis-*associated virulence factor genes (*PPAD*, *RgpA* and *RgpB*) and adhesive molecular genes (Fimbriae *fimA*, hemagglutinins *HagA* and *HagB*) 17,45 were evaluated. As shown in **Figure 6A,** key toxins of *P. gingivalis*, such as *FimA*, *RgpA*, *RgpB*, *PPAD*, *HagA* and *HagB*, were positively downregulated in the Ag_2_S+NIR and Ag_2_S@ZIF-90+NIR groups under temperature stress. These results indicated that heat exerted a certain effect on reducing *P. gingivalis* virulence factors and adhesion molecules in biofilms. It has been demonstrated that lower temperature was beneficial to the activity of the *fimA* promoter, while higher temperature weakened the hemagglutination activity, affecting the adhesion regulatory virulence of bacteria 17. The results showed that the photothermal treatment groups could effectively inhibit the expression of adhesion genes and virulence factors, thus hindering bacterial colonization and interfering with the interaction between bacteria and host. However, the expression of related factors in the Ag_2_S@ZIF-90/ICG+NIR and Ag_2_S@ZIF-90/Arg/ICG+NIR groups with heat, ROS and NO decreased significantly, especially in the Ag_2_S@ZIF-90/Arg/ICG+NIR group. In this process, nitrosation stress or oxidative stress plays a leading role. The oxidative stress caused by ROS causes irreversible damage to DNA and proteins. At the same time, nitrosation stress can lead to DNA deamination on proteins and thiol nitrosation. In conclusion, NCs with aPTT/aPDT/NO capacity could effectively reduce bacterial invasiveness and inhibit the expression of bacterial virulence factors, which further regulated the local periodontal microenvironment and was of great significance for the treatment of periodontitis.

Motivated by the excellent *in vitro* anti-biofilm effect and the decreased virulence factor of *P. gingivalis*, further experiments were conducted to explore the internal mechanism of anti-biofilm effects by nanocomposites. Recent work indicated that c-di-AMP was the major and critical signaling system in *P. gingivalis* 46, controlling cell proliferation, biofilm formation, and virulence. Through previous bioinformatics analysis, there was a c-di-AMP synthetase-encoding gene (*pgn 0523*) 44 and three c-di-AMP catabolic enzyme-encoding genes (*pgn 1187*, *pgn 2003*, and *pgn 521*) 47 in the genome of *P. gingivalis.* In groups with aPTT effects (Ag_2_S+NIR and Ag_2_S@ZIF-90+NIR)**,** the expression of *pgn 0523, pgn 1187, pgn 2003* and *pgn 0521* was almost identical to that of the control group (**Figure 6B**). It was reported that the CFU counts of the mutant lacking c-di-AMP synthetase and lacking c-di-AMP hydrolase of *Streptococcus mitis* were not different from that of wild-type (WT) at 52 °C, demonstrating that there were scarcely any effects of temperature alteration on c-di-AMP expression 48. In the Ag_2_S@ZIF-90/ICG+NIR group, the expression level of *pgn 0523* was approximately 3-fold higher than that of the control since biofilms enhance the response to ROS pressure by synthesizing larger amounts of c-di-AMP. Although c-di-AMP is vital for biofilm metabolism and bacterial growth, superfluous accumulation of c-di-AMP is deleterious for bacterial cell functions 49. Correspondingly, the expression levels of *pgn 1187, pgn 2003* and* pgn 0521* were also increased to balance the c-di-AMP level in the biofilm. Interestingly, *in vitro* anti-biofilm experiments (**Figure 5**) showed that the experimental group with aPTT and aPDT had adequate bactericidal effects, which suggested that the change in c-di-AMP concentration was not the only determinant of anti-biofilm. Due to the explosive ROS generation of aPDT, a great deal of ROS directly kill pathogens by damaging bacterial cell membranes and DNA. Remarkably, the expression levels of *pgn 1187*, *pgn 2003* and* pgn 0521* were largely increased in Ag_2_S@ZIF-90/Arg/ICG+NIR with NO production. Previous reports suggested that the binding of heme to the domain would inhibit PDE activity to further result in the accumulation of c-di-AMP 15. However, NO production could competitively coordinate with heme to the domain, thus stimulating PDE activity to accelerate the degradation of c-di-AMP accordingly 50. In summary, as illustrated in **Figure 6C**, NO could activate PDE activity to cause c-di-AMP degradation and then lead to biofilm decomposition. As a result, the contact between the NCs and the bacteria was greatly increased, producing a significant anti-biofilm effect. More importantly, the biofilm dispersed with the help of heat did not become denser, which was beneficial for improving the penetrability of ROS and resulted in outstanding antibiofilm performance.

### *In vivo* anti-biofilm performance

**Figure 7:**
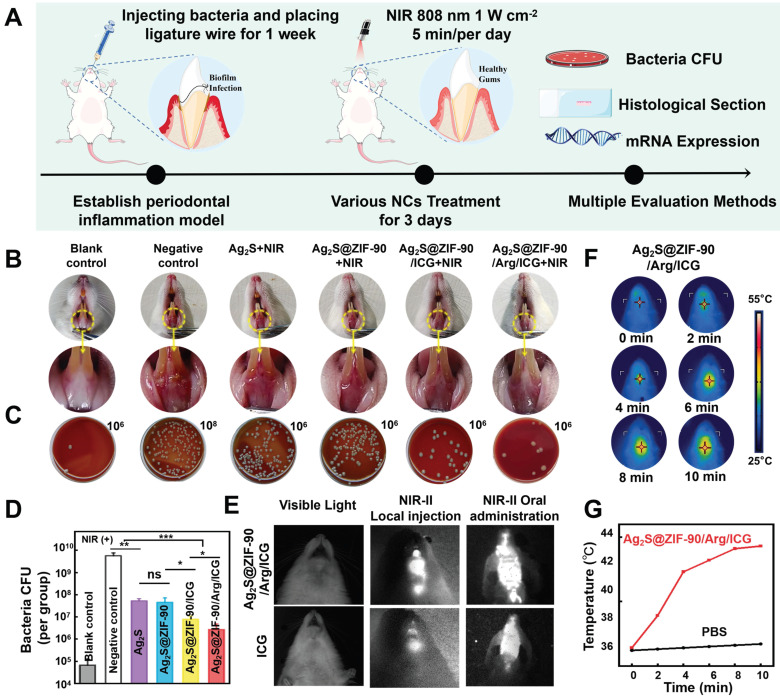
***In vivo* antibiofilm effect of different NCs.** (A) Illustration diagram of *in vivo* anti-biofilm treatment of periodontal inflammation. (B) Intraoral photos. (C) Colonies isolated from gingival tissue represent the antimicrobial function of different NCs in rats *in vivo*. The dilution factors were set in the upper right portion of the blood agar plate. (D) Corresponding bar graph of colonies. (E) Ag_2_S@ZIF-90/Arg/ICG NCs and ICG imaging of rats in the NIR II region. (F) Photographs of photothermal effects of Ag_2_S@ZIF-90/Arg/ICG NCs* in vivo* under 808 nm NIR excitation and (G) Relevant temperature curve record plot. (n = 3, *p < 0.05, **p < 0.01, ***p < 0.001, ns, not significant.)

Finally, the antibiofilm effects of different NCs were investigated in animal models with periodontal inflammation infected by periodontal pathogens. The entire treatment procedure for periodontal inflammation in rats is summarized in **Figure 7A.** Briefly, to establish rat models of periodontitis, the anterior mandibular teeth were ligated at the neck margin, and a mixed bacterial solution was injected. After 7 days, different nanocomposites were irradiated with 808 nm NIR for 3 consecutive days. As shown in **Figure 7B**, the gingival tissue of rats in the negative control group was dark red, and white abscess spots appeared in part of the mucosa, demonstrating severe local periodontal inflammation without any treatments. Compared with the negative control group, the gingival inflammation in the Ag_2_S+NIR and Ag_2_S@ZIF-90+NIR groups was slightly relieved, and the gingival status of the Ag_2_S@ZIF-90/Arg/ICG+NIR group improved significantly, almost returning to normal. The gingival status of Ag_2_S@ZIF-90/ICG+NIR was intermediate between the groups with aPTT and the group with aPTT/aPDT/NO. As shown in **Figure 7C-7D**, compared to the negative control group, the CFU of the Ag_2_S@ZIF-90/Arg/ICG group decreased by nearly 3 log. Notably, affected by the inherent microbiota of the oral cavity and the nonsterile environment in the rat mouth, the antibacterial effect *in vivo* is not as obvious as that *in vitro*. Unfortunately, no relief of inflammation in the experimental groups without 808 nm NIR was observed (**Figure S22A**). Similarly, the CFU of their gingival tissue did not change significantly compared with that of the negative control group (**Figure S22B-S22C**). Combined with the above results, the relief of local inflammation may be because the reduction in local bacterial colonies would further decrease the stimuli of c-di-AMP. Changes in the concentrations of c-di-AMP could alter bacterial pathogenicity in mouse models and thus participate in the regulation of virulence *in vivo* 51,52. In addition, several receptors of the innate immune system can recognize c-di-AMP, which induces NF-κB activation or type I interferon production 17,48. This was also demonstrated by *in vitro* experiments simulating inflammation caused by biofilm infection (**Figure S23**). Coincidentally, our previous study confirmed that exogenous NO could inhibit the NF-κB pathway by inducing S-nitrification of IKKβ and p65 17. Combined with the aforementioned results in **Figure 6B**, NO could stimulate the c-di-AMP-catabolic enzyme PDE, thus reducing the content of c-di-AMP to further alleviate local inflammation. Therefore, it was speculated that Ag_2_S@ZIF-90/Arg/ICG+NIR would effectively reduce c-di-AMP levels and inhibit the NF-κB pathway, which ultimately relieved inflammation. ICG has been extensively applied to medical fields such as measuring cardiac output, monitoring liver function, and ophthalmic angiography 53. Both local injection and oral administration 54 are shown in **Figure 7E**, and bright areas were confined to the injection site or to the mouse mouth. This suggests that Ag_2_S@ZIF-90/Arg/ICG NCs had the same imaging function as ICG in the near infrared region II (NIR-II).

In particular, oral administration in the form of mouthwash also had excellent NIR-II imaging function, and the outline of the posterior wall of the oropharynx in mice was visible. This noninvasive method not only reduced biological suffering but also provided a new promising option for dental imaging in the future. The *in vivo* photothermal effect of the Ag_2_S@ZIF-90/Arg/ICG NCs group is depicted in **Figure 7F-7G**. The temperature at the gingival site of the rats in the Ag_2_S@ZIF-90/Arg/ICG NCs group rose rapidly to 44.3 °C after ten minutes of NIR irradiation, suggesting that the Ag_2_S@ZIF-90/Arg/ICG NCs were also able to respond to local surface plasmon resonances triggered by near-infrared light *in vivo*. Mild thermal therapy was reported to be beneficial for bacterial biofilm dispersal and killing but without any damage to normal tissues 55.

**Figure 8:**
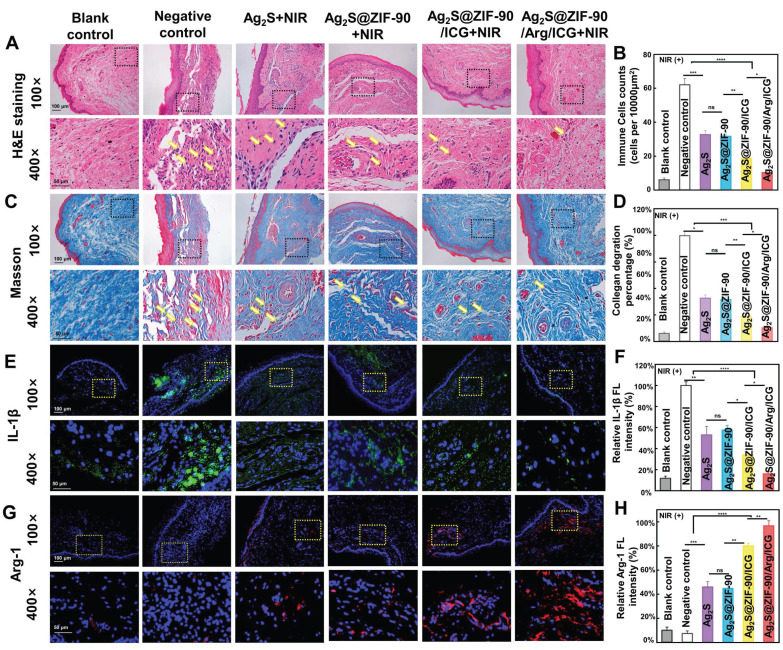
***In vivo* histomorphological evaluation of inflammation of the periodontal tissue after various NC treatments under 808 nm NIR irradiation.** (A) Typical H&E staining images (yellow arrows: inflammatory cells) and (B) statistics of the number of immune cells at the inflammatory site of gingival tissue. (C) Typical Masson's staining images (yellow arrows: degraded collagen) and (D) percentage of collagen degradation at the inflammatory site of gingival tissue. (E) Immunofluorescence images of gingival tissue (green fluorescence represents IL-1β-positive cells, and blue fluorescence represents nuclei). (G) Immunofluorescence images of gingival tissue (red fluorescence represents Arg-1-positive cells, and blue fluorescence represents nuclei). The corresponding immunofluorescence intensity of IL-1β (F) and Arg-1 (H) was quantified. (n = 3, *p < 0.05, **p < 0.01, ***p < 0.001, ****p < 0.0001, ns, not significant.)

The tissue around periodontal inflammation was harvested for histological evaluation. First, the density of inflammatory cells in gingival tissue was analyzed using H&E staining (**Figure 8A**). In the negative control group and the groups without 808 nm NIR stimulation (**Figure S24**), the inflammatory cells gathered in clumps. In all groups with 808 nm NIR irradiation, there was a substantial decrease in the count of inflammatory cells gathering at the site of inflammation. According to **Figure 8B**, the Ag_2_S@ZIF-90/Arg/ICG+NIR group had the lowest number of inflammatory cells, indicating that NO could not only kill bacteria with the synergistic function of aPTT and aPDT but also act as an inflammatory regulator to regulate immune responses. When a bacterium is killed, its remains, such as DNA, ATP and other damage-related molecular patterns (DAMPs), also trigger a further inflammatory response 56. In contrast, the DAMP-induced immune response and inflammatory injury can be regulated and alleviated by NO 17. Moreover, it has been reported that NO can regulate neutrophil migration and coordinate leukocyte recruitment during inflammation 57. Thus, NO-assisted aPTT and aPDT could be helpful for combating periodontal pathogens and modulating local inflammation.

Second, the degradation of collagen fibers was investigated in the inflammatory site by Masson staining (**Figure 8C-8D**), since collagen fibers in periodontal tissue can be degraded by collagenase generated by pathogenic bacteria. Dense and orderly arranged collagen fibers (blue staining) were observed in the blank control. However, in the experimental group without 808 nm NIR treatment (**Figure S25**), collagen fiber arrangement disorder and fragmentation occurred under the infiltration of inflammatory cells. Upon 808 nm NIR irradiation, different groups exhibited collagen degradation to different degrees. Ag_2_S+NIR and Ag_2_S@ZIF-90+NIR slightly hindered the degradation of collagen with mild thermal effects. When aPDT and enhanced aPTT were involved, Ag_2_S@ZIF-90/ICG had a significant reduction in collagen degradation. The NO-assisted aPTT and aPDT (Ag_2_S@ZIF-90/Arg/ICG+NIR group) possessed the lowest rate of collagen degradation (**Figure 8C-8D**). The main reason was probably that type I collagen is the main collagen fiber of the periodontal ligament, and exogenous NO could stimulate the expression of type I collagen, thus compensating for the degradation of collagen caused by bacteria-induced infections 57.

Third, immunofluorescence staining and RT‒qPCR were used to detect the expression of proinflammatory and anti-inflammatory factors. Inflammation caused by bacterial infection is actually a physiological response to stimuli, which is generally induced by inflammatory cytokines. In inflammatory diseases caused by pathogens, macrophages, as heterogeneous immune cells, can be polarized into proinflammatory M1 and anti-inflammatory M2 phenotypes according to different microenvironments 58. As shown by immunofluorescence staining (**Figure 8E**-**8H)**, the experimental groups under 808 nm NIR irradiation showed decreased expression of the proinflammatory mediator IL-1β and increased expression of the anti-inflammatory mediator Arg-1. Among them, Ag_2_S@ZIF-90/Arg/ICG+NIR showed the slightest expression of IL-1β and the highest expression of Arg-1. The balanced expression of proinflammatory and anti-inflammatory factors in inflammatory tissues, including inhibition of M1 polarization and enhancement of M2 polarization in macrophages, demonstrated the potential inflammatory regulatory effects of Ag_2_S@ZIF-90/Arg/ICG+NIR. It has been reported that in the presence of exogenous NO, stem cells release proregenerative and immunomodulatory factors for wound repair, and keratinocytes can also proliferate and differentiate to accelerate wound healing and epithelial formation 59. Furthermore, the genetic level of inflammation (**Figure S27**)-related biomarkers, including IL-1β and Arg-1, showed a similar trend as the protein expression in immunofluorescence staining. In contrast, immunofluorescence staining (**Figure S26**) and real-time PCR (**Figure S28**) of the gingival tissues in the non-808 nm NIR irradiation groups were not remarkably different from those in the negative control group.

### Biosafety assessment of Ag_2_S@ZIF-90/Arg/ICG

To evaluate the utility and feasibility of Ag_2_S@ZIF-90/Arg/ICG NCs for clinical treatment, the *in vitro* and* in vivo* biosafety was finally verified 60. A CCK8 kit was utilized to investigate the *in vitro* safety of NCs in the mouse fibroblast cell Line L929 for one day and three days. The results showed that when the concentration was less than or equal to 0.092 mM, the cell survival rate was above 90% **(Figure S29A-S29B)**. When the NCs content increased to 0.184 mM, the viability of the cells decreased obviously to approximately 70%. Meanwhile, real-time monitoring of cell growth showed a growth curve for Ag_2_S@ZIF-90/Arg/ICG NCs at concentrations below 0.092 mM similar to that of the control group (**Figure S29C**), proving the excellent biocompatibility of Ag_2_S@ZIF-90/Arg/ICG NCs. As visually presented in **Figure S29D,** when the concentration of Ag_2_S@ZIF-90/Arg/ICG NCs was lower than 0.092 mM, DAPI-labeled nuclei in human gingival fibroblasts showed blue fluorescence and were uniform and intact without apoptosis. While few nuclei were concentrated, the cell morphology became round and wrinkled at a Ag_2_S@ZIF-90/Arg/ICG NCs concentration of 0.184 mM. The *in vivo* toxicity was appraised by histological examination of five major organs: heart, liver, spleen, lung and kidney, and no evident histological variations were found, indicating that the different NCs had good* in vivo* biosafety (**Figure S29E**). **Figure S29F** shows that the hemolytic rate of NCs concentrations below 0.184 mM was less than 5%, indicating that Ag_2_S@ZIF-90/Arg/ICG NCs had favorable blood compatibility. Hence, the low concentration (below 0.092 mM) of Ag_2_S@ZIF-90/Arg/ICG NCs possessed excellent biocompatibility and was not harmful for clinical application.

## Conclusions

Nanoparticle-based NO gas delivery systems have demonstrated excellent antibiofilm performance to meet the challenges posed by biofilm infections. However, the current popular NO delivery systems, the unstable release of NO, the high cost of synthesis and the toxicity of byproducts hinder the application of such nanoparticles. Therefore, in this work, core-shell Ag_2_S@ZIF-90 NCs were developed, and then their surface was loaded with L-arg and ICG. The designed Ag_2_S@ZIF-90/Arg/ICG NCs possessed photothermal effects and produced overwhelming amounts of ROS with 808 nm NIR laser excitation. NO molecules were simultaneously generated by the oxidation of L-arg in the presence of ROS. By such a design, favorable antibiofilm efficiency was realized by synergistic therapy with aPTT/aPDT and NO production. The CFU counts of periodontal pathogens showed a 4-log reduction with the NO-complemented aPTT/aPDT treatments. More importantly, the underlying mechanisms of biofilm dispersal were explored, and NO could upregulate the genetic level of c-di-AMP catabolic enzymes to activate PDE activity in mature biofilms and thus decompose c-di-AMP. Once biofilm was dispersed, heat and ROS could quickly kill bacteria free from biofilm. In animal models of periodontal diseases, Ag_2_S@ZIF-90/Arg/ICG NCs possessed the capacity to alleviate periodontal inflammation by dissipating biofilm. In addition, nanocomposites with NO-complemented aPTT/aPDT effects could decrease local inflammatory cells, prevent collagen degradation, and regulate inflammatory mediators for tissue repair. Overall, this study provides a strategy for the clinical treatment of periodontal disease from the perspective of biofilm elimination and could probably be extended to other intractable bacteria-infected diseases.

## Supplementary Material

Supplementary methods, figures and tables.

## References

[1] Kinane DF, Stathopoulou PG, Papapanou PN (2017). Periodontal diseases. Nat Rev Dis Primers.

[2] Di Stefano M, Polizzi A, Santonocito S, Romano A, Lombardi T, Isola G (2022). Impact of oral microbiome in periodontal health and periodontitis: a critical review on prevention and treatment. Int J Mol Sci.

[3] Cobb CM, Sottosanti JS (2021). A re-evaluation of scaling and root planing. J Periodontol.

[4] Czesnikiewicz Guzik M, Osmenda G, Siedlinski M, Nosalski R, Pelka P, Nowakowski D (2019). Causal association between periodontitis and hypertension: evidence from mendelian randomization and a randomized controlled trial of non-surgical periodontal therapy. Eur Heart J.

[5] Larsson D, Flach CF (2022). Antibiotic resistance in the environment. Nat Rev Microbiol.

[6] Szabo C (2016). Gasotransmitters in cancer: from pathophysiology to experimental therapy. Nat Rev Drug Discov.

[7] Peña CAP, González R, López-Grueso MJ, Antonio Bárcena J (2015). Redox. Regulation of metabolic and signaling pathways by thioredoxin and glutaredoxin in nitric oxide treated hepatoblastoma cells. Redox Biol.

[8] Hays E, Bonavida B (2019). Nitric oxide-mediated enhancement and reversal of resistance of anticancer therapies. Antioxidants.

[9] Fan W, Lu N, Huang P, Liu Y, Yang Z, Wang S (2017). Glucose-responsive sequential generation of hydrogen peroxide and nitric oxide for synergistic cancer starving-like/gas therapy. Angew Chem Int Edit.

[10] Francés R, Muñoz C, Zapater P, Uceda F, Gascón I, Pascual S (2004). Bacterial DNA activates cell mediated immune response and nitric oxide overproduction in peritoneal macrophages from patients with cirrhosis and ascites. Gut.

[11] Hossain S, Nisbett LM, Boon EM (2017). Discovery of two bacterial nitric oxide-responsive proteins and their roles in bacterial biofilm regulation. Acc Chem Res.

[12] Rumbaugh KP, Sauer K (2020). Biofilm dispersion. Nat Rev Microbiol.

[13] Meng Z, Wei F, Ma W, Yu N, Wei P, Wang Z (2016). Design and synthesis of “all-in-one” multifunctional FeS_2_ nanoparticles for magnetic resonance and near-infrared imaging guided photothermal therapy of tumors. Adv Funct Mater.

[14] Yang T, Wang Y, Ke H, Wang Q, Lv X, Wu H (2016). Protein-nanoreactor-assisted synthesis of semiconductor nanocrystals for efficient cancer theranostics. Adv Mater.

[15] Corrigan RM, Gründling A (2013). Cyclic di-AMP: another second messenger enters the fray. Nat Rev Microbiol.

[16] Wang Z, Zhan M, Li W, Chu C, Xing D, Lu S (2021). Photoacoustic cavitation-ignited reactive oxygen species to amplify peroxynitrite burst by photosensitization-free polymeric nanocapsules. Angew Chem Int Edit.

[17] Qi M, Ren X, Li W, Sun Y, Sun X, Li C (2022). NIR responsive nitric oxide nanogenerator for enhanced biofilm eradication and inflammation immunotherapy against periodontal diseases. Nano Today.

[18] Zheng J, Wang W, Gao X, Zhao S, Chen W, Li J (2022). Cascade catalytically released nitric oxide-driven nanomotor with enhanced penetration for antibiofilm. Small.

[19] Riccio DA, Schoenfisch MH (2012). Nitric oxide release: part I. macromolecular scaffolds. Chem Soc Rev.

[20] Zheng DW, Chen Y, Li ZH, Xu L, Li CX, Li B (2018). Optically-controlled bacterial metabolite for cancer therapy. Nat Commun.

[21] Jiang Z, Dong B, Chen B, Wang J, Xu L, Zhang S (2013). Multifunctional Au@mSiO_2_/rhodamine B isothiocyanate nanocomposites: cell imaging, photocontrolled drug release, and photothermal therapy for cancer cells. Small.

[22] Zhang Z, Wang L, Wang J, Jiang X, Li X, Hu Z (2012). Mesoporous silica-coated gold nanorods as a light-mediated multifunctional theranostic platform for cancer treatment. Adv Mater.

[23] He J, Ramachandraiah K, Huang T, Yuan T, Liu X, Zhang H (2023). Core-shell structured hollow copper sulfide@metal-organic framework for magnetic resonance imaging guided photothermal therapy in second near-infrared biological window. Biochem Biophys Res Commun.

[24] Ye Z, Jiang Y, Li L, Wu F, Chen R (2021). Rational design of MOF-based materials for next-generation rechargeable batteries. Nanomicro Lett.

[25] Jiang Z, Wang Y, Sun L, Yuan B, Tian Y, Xiang L (2019). Dual ATP and pH responsive ZIF-90 nanosystem with favorable biocompatibility and facile post-modification improves therapeutic outcomes of triple negative breast cancer *in vivo*. Biomaterials.

[26] Lv L, Wang H (2014). Ag_2_S nanorice: hydrothermal synthesis and characterization study. Mater Lett.

[27] Nosike EI, Jiang Z, Miao L, Akakuru OU, Yuan B, Wu S (2020). A novel hybrid nanoadsorbent for effective Hg^2+^ adsorption based on zeolitic imidazolate framework (ZIF-90) assembled onto poly acrylic acid capped Fe_3_O_4_ nanoparticles and cysteine. J Hazard Mater.

[28] Yuan Z, Lin C, He Y, Tao B, Chen M, Zhang J (2020). Near-infrared light-triggered nitric-oxide-enhanced photodynamic therapy and low-temperature photothermal therapy for biofilm elimination. ACS Nano.

[29] Liu J, Kong T, Xiong HM (2022). Mulberry-leaves-derived red-emissive carbon dots for feeding silkworms to produce brightly fluorescent silk. Adv Mater.

[30] Han R, Xiao Y, Yang Q, Pan M, Hao Y, He X (2021). Ag_2_S nanoparticle-mediated multiple ablations reinvigorates the immune response for enhanced cancer photo-immunotherapy. Biomaterials.

[31] Mei D, Liu L, Li H, Wang Y, Ma F, Zhang C (2022). Efficient uranium adsorbent with antimicrobial function constructed by grafting amidoxime groups on ZIF-90 via malononitrile intermediate. J Hazard Mater.

[32] Morris W, Doonan CJ, Furukawa H, Banerjee R, Yaghi OM (2008). Crystals as molecules: postsynthesis covalent functionalization of zeolitic imidazolate frameworks. J Am Chem Soc.

[33] Wang H, Li X, Tse BW-C, Yang H, Thorling CA, Liu Y (2018). Indocyanine green-incorporating nanoparticles for cancer theranostics. Theranostics.

[34] Li W, Zhang J, Gao Z, Qi J, Ding D (2022). Advancing biomedical applications via manipulating intersystem crossing. Coordin Chem Rev.

[35] Kuo W-S, Chang Y-T, Cho KC, Chiu KC, Lien CH, Yeh CS (2012). Gold nanomaterials conjugated with indocyanine green for dual-modality photodynamic and photothermal therapy. Biomaterials.

[36] Zhu J, Tian J, Yang C, Chen J, Wu L, Fan M (2021). L-arg-rich amphiphilic dendritic peptide as a versatile NO donor for NO/photodynamic synergistic treatment of bacterial infections and promoting wound healing. Small.

[37] Xu T, Dong Q, Luo Y, Liu Y, Gao L, Pan Y (2021). Porphyromonas gingivalis infection promotes mitochondrial dysfunction through Drp1-dependent mitochondrial fission in endothelial cells. Int J Oral Sci.

[38] Brennan CA, Garrett WS (2019). Fusobacterium nucleatum-symbiont, opportunist and oncobacterium. Nat Rev Microbiol.

[39] Xie J, Zhou M, Qian Y, Cong Z, Chen S, Zhang W (2021). Addressing MRSA infection and antibacterial resistance with peptoid polymers. Nat Commun.

[40] Cai X, Tian J, Zhu J, Chen J, Li L, Yang C (2021). Photodynamic and photothermal co-driven CO-enhanced multi-mode synergistic antibacterial nanoplatform to effectively fight against biofilm infections. Chem Eng J.

[41] Sun L, Jiang W, Zhang H, Guo Y, Chen W, Jin Y (2018). Photosensitizer-loaded multifunctional chitosan nanoparticles for simultaneous *in situ* imaging, highly efficient bacterial biofilm eradication, and tumor ablation. ACS Appl Mater.

[42] Huo J, Jia Q, Huang H, Zhang J, Li P, Dong X (2021). Emerging photothermal-derived multimodal synergistic therapy in combating bacterial infections. Chem Soc Rev.

[43] Awwad AM, Salem NM, Aqarbeh MM, Abdulaziz FM (2020). Green synthesis, characterization of silver sulfide nanoparticles and antibacterial activity evaluation. Chem Int.

[44] Moradali MF, Ghods S, Bähre H, Lamont RJ, Scott DA, Seifert R (2022). Atypical cyclic di-AMP signaling is essential for porphyromonas gingivalis growth and regulation of cell envelope homeostasis and virulence. NPJ Biofilms Microbi.

[45] Darveau RP (2010). Periodontitis: a polymicrobial disruption of host homeostasis. Nat Rev Microbiol.

[46] Xia P, Wang S, Xiong Z, Zhu X, Ye B, Du Y (2018). The ER membrane adaptor ERAdP senses the bacterial second messenger c-di-AMP and initiates anti-bacterial immunity. Nat Immunol.

[47] Wei Q, Xingqun C, Xuedong Z, Yuqing L (2015). Cloning, expression, and purification of c-di-AMP metabolism-related genes from porphyromonas gingivalis. Hua xi kou Qiang yi xue za zhi.

[48] Rørvik GH, Naemi AO, Edvardsen PKT, Simm R (2021). The c-di-AMP signaling system influences stress tolerance and biofilm formation of streptococcus mitis. MicrobiologyOpen.

[49] Xiong ZQ, Fan YZ, Song X, Liu XX, Xia YJ, Ai LZ (2020). The second messenger c-di-AMP mediates bacterial exopolysaccharide biosynthesis: a review. Mol Med Rep.

[50] Christen M, Christen B, Folcher M, Schauerte A, Jenal U (2005). Identification and characterization of a cyclic di-GMP-specific phosphodiesterase and its allosteric control by GTP. J Biol Chem.

[51] Bai Y, Yang J, Eisele LE, Underwood AJ, Koestler BJ, Waters CM (2013). Two DHH subfamily 1 proteins in streptococcus pneumoniae possess cyclic di-AMP phosphodiesterase activity and affect bacterial growth and virulence. J Bacteriol.

[52] Hu J, Zhang G, Liang L, Lei C, Sun X (2020). Increased excess intracellular cyclic di-AMP levels impair growth and virulence of bacillus anthracis. J Bacteriol.

[53] Yannuzzi LA (2011). Indocyanine green angiography: a perspective on use in the clinical setting. Am J Ophthalmol.

[54] Li Z, Li Z, Zaid W, Osborn ML, Li Y, Yao S (2022). Mouthwash as a non-invasive method of indocyanine green delivery for near-infrared fluorescence dental imaging. J Biomed Opt.

[55] Li Y, Liu X, Cui Z, Zheng Y, Jiang H, Zhang Y (2022). Treating multi-drug-resistant bacterial infections by functionalized nano-bismuth sulfide through the synergy of immunotherapy and bacteria-sensitive phototherapy. ACS Nano.

[56] An P, Wei LL, Zhao S, Sverdlov DY, Vaid KA, Miyamoto M (2020). Hepatocyte mitochondria-derived danger signals directly activate hepatic stellate cells and drive progression of liver fibrosis. Nat Commun.

[57] Malone-Povolny MJ, Maloney SE, Schoenfisch MH (2019). Nitric oxide therapy for diabetic wound healing. Adv Healthc Mater.

[58] Gu Q, Yang H, Shi Q (2017). Macrophages and bone inflammation. J Orthop Translat.

[59] Miyamoto T, Petrus MJ, Dubin AE, Patapoutian A (2011). TRPV3 regulates nitric oxide synthase-independent nitric oxide synthesis in the skin. Nat Commun.

[60] Sun X, Sun J, Sun Y, Li C, Fang J, Zhang T (2021). Oxygen self-sufficient nanoplatform for enhanced and selective antibacterial photodynamic therapy against anaerobe-induced periodontal disease. Adv Funct Mater.

