# Supplementary material for: Near‐infrared light-triggered nitric oxide nanocomposites for photodynamic/photothermal complementary therapy against periodontal biofilm in an animal model
Source: Theranostics. 2023 Apr 17;13(7):2350–67. doi: 10.7150/thno.83745 (PMC10157734; doi:10.7150/thno.83745)
Supplement: Supplementary file 1 — Supplementary methods, figures and tables. [file thnov13p2350s1.pdf]

Supporting information

Near-infrared light triggered nitric oxide nanocomposites for photodynamic/photothermal complementary therapy against periodontal biofilm in animal model

Xiangrong Wu[1], Manlin Qi[1], Chengyu Liu[1], Qijing Yang[1], Sijia Li[1], Fangyu Shi[1], Xiaolin Sun[1], Lin Wang[1*], Chunyan Li[1]*, Biao Dong[2]*

[1]*Department of Prosthodontics, Jilin Provincial Key Laboratory of Tooth Development and Bone Remodeling, School and Hospital of Stomatology, Jilin University, Changchun, 130021, P. R. China;*

[2]*State Key Laboratory on Integrated Optoelectronics, College of Electronic Science and Engineering, Jilin University, Changchun, 130012, P. R. China*

***Correspondence:**

Prof. Chunyan Li, Email: cyli@jlu.edu.cn

Prof. Biao Dong, Email: dongb@jlu.edu.cn

Prof. Lin Wang, Email: wanglin1982@jlu.edu.cn

1. Additional Experimental Methods

1.1 Materials

Chemicals: Imidazole-2-formaldehyde (2-ICA) and indocyanine green (ICG) were purchased from Macklin Inc (Shanghai, China). Silver nitrate ($AgNO_3$), Sodium sulfide hydrate ($Na_2S\cdot9H_2O$), Zinc acetate dihydrate ($Zn(CH_3COO)_2.2H_2O$, ethanol and methanol were purchased from Beijing Chemical Works (Beijing, China). The 1,3-diphenylisobenzo-furan (DPBF), N,N-Dimethylformamide (DMF), polyvinyl pyrrolidone (PVP) polyacrylic acid (PAA), brain heart infusion medium (BHI) and L-Arginine (L-arg) were purchased from Aladdin (Shanghai, China). 9,10-anthracenedipropanoic acid (ABDA) and 2',7'-Dichlorodihydrofluorescein diacetate (DCFH-DA) were bought from Sigma-Aldrich (St. Louis, Missouri, USA). Nitric Oxide Detection assay kit and H_2O_2 standard substance were purchased from Biyuntian Biotechnology Co., LTD (Shanghai, China). Amino acid detection kit was purchased from Yuanye biotechnology Co., LTD (Shanghai, China). Dulbecco's Modified Eagle Medium (DMEM) and fetal bovine serum (FBS) were obtained from Gibco (Grand Island, NY, USA). Penicillin-Streptomycin Solution was purchased from Hyclone (GE Healthcare, Logan, UT, USA). Fluorescein isothiocyanate (FITC), and 4', 6-diamidino-2-phenylindole (DAPI) were purchased from Sigma-Aldrich (St. Louis, MO, USA). LIVE/DEAD BacLight Bacterial Viability Kit was purchased from Thermo Fisher Scientific to Acquire PPD, Inc, China. RNAiso plus, PrimeScript RT Reagent Kit with gDNA Eraser, and TB Green Premix *Ex Taq II* were purchased from Takara Bio Inc (Kusatsu, Shiga, Japan). Tryptic soy broth (BHI), menadione and hemin were purchased from Sigma-Aldrich (St. Louis, MO, USA). Columbia blood agar was purchased from BIO-KONT (Wenzhou, China). NF-κB/p65 primary antibodies(F-6): sc-8008 was purchased from Santa Cruz Biotechnology, Inc (Shanghai, China). Goat Anti-Mouse IgG H&L (Alexa Fluor® 647) secondary antibodies were obtained from Abcam (Cambridge, MA, USA)

1.2 Preparation of Ag_2S@ZIF-90/Arg/ICG

Ag_2S NPs were synthesized according to the previous work [1]. Briefly, 240 mg of $Na_2S\cdot9H_2O$ and 350 mg of PVP were dissolved with 10 mL of deionized water under magnetic stirring. The 10 mL $AgNO_3$ solution (0.1 M) was added dropwise to the above liquids. After continuous stirring for 2 h, the mixture solution was then transferred to a 25 mL Teflon-coated high-pressure tank and maintained at 160 °C for 5 h. Afterwards, the tank was cooled to room temperature. Ag_2S NPs were obtained by centrifugation (12000 rmp, 15 min) and washed three times with methanol and ethanol.

Then Ag_2S NCs were dispersed in 10 mL methanol for further use.

Subsequently, PAA was coated onto the surface of Ag_2S NPs [2]. First, 3 mL of PAA (0.223 g mL^{-1}) methanol solution was added into 10 mL of Ag_2S NPs methanol solution (0.01 M) followed by stirring and ultrasonic dispersal. After continuous magnetic stirring for 24 h, Ag_2S@PAA NCs were separated by centrifugation (12,000 rmp, 15min), and washed with methanol and DMF for three times. Then Ag_2S@PAA NCs were stored in DMF.

Ag_2S@ZIF-90 NCs were synthesized as described in previous studies [2]. At first, 10 mL DMF solution containing $Zn(CH_3COO)_2 \cdot 2H_2O$ (0.04 M) was continuously stirred with Ag_2S@PAA NCs (0.01M) solution for 5 hours. Thereafter, 10 mL of ICA (0.02 M) solution was added quickly to the above mixture for 1 h with vigorous stir. The products were washed three times with DMF and DI water. The obtained Ag_2S@ZIF-90 NCs were dispersed in 10 mL DI water for further use.

To immobilize L-arg molecules in the pores of Ag_2S@ZIF-90 NCs [3], 20 mL L-arg (0.01M) DI water solution and 10 mL Ag_2S@ZIF-90 NCs solution were stirred at room temperature for 4 h. Ag_2S@ZIF-90/Arg NCs were obtained by centrifugation and washed with DI water for three times.

ICG (0.357 mg mL^{-1} 10 mL) DI water solution was added into Ag_2S@ZIF-90/Arg NCs (0.01 M 10 mL) DI water solution. The mixture was stirred overnight at room temperature. Similarly, ICG-loaded Ag_2S@ZIF-90 NCs (Ag_2S@ZIF-90/ICG NCs) were also prepared via the above method.

1.3 ICG loading efficacy

To evaluate the drug-loading effect of ICG, the resulting Ag_2S@ZIF-90/Arg/ICG NCs and the supernatant containing unloaded ICG were collected by centrifugation. Spectra of both pure ICG gradient solution (15 μg mL^{-1}, 30 μg mL^{-1}, 60 μg mL^{-1}, 120 μg mL^{-1}, 240 μg mL^{-1}) and supernatant solution were determined by UV-vis. The concentration of unloaded ICG was calculated using a fitted standard curve of pure ICG. UV−vis spectra of free ICG with different concentrations and first centrifugal fluid of Ag_2S@ZIF-90/Arg/ICG NCs were depicted in **figure S5**. The loading capacity and encapsulation efficiency of ICG were calculated using the following formula:

loading capacity (w/w %) = (ICG loading weight/final NCs weight) × 100%.

Trapping efficiency (w/w %) = (ICG loading weight/ICG input weight) × 100%.[4]

1.4 L-arg loading efficacy

The resulting Ag_2S@ZIF-90/Arg NCs and the supernatant containing unloaded L-arg were collected by centrifugation. Under the condition of heating and weak acid, the α-amino group in the

free amino acid can react with ninhydrin hydrate to form a blue-purple compound. The product has a characteristic absorption peak at 570 nm. The amino acid content can be detected quantitatively by the change of the absorption value. The concentration of unloaded L-arg was calculated using a fitted standard curve.

loading capacity (w/w %) = (L-arg loading weight/Ag_2S@ZIF-90/Arg NCs weight) ×100%.

Trapping efficiency (w/w %) = (L-arg loading weight/L-arg input weight) × 100%.

1.5 Photothermal performance of Ag_2S@ZIF-90/Arg/ICG NCs

To measure *in vitro* photothermal effects, Ag_2S@ZIF-90/Arg/ICG NCs were placed in a 1.5 ml EP tube and then irradiated by an 808 nm laser (BWT Beijing Ltd., DS3-11313-0411, max: 5W,10,000 Hz, 45 μs) with different power densities (0.5, 1.0, 2.0 W cm^{-2}) for 10 min. Besides, different concentrations (0.046 mM, 0.092 mM, and 0.184 mM) of Ag_2S@ZIF-90/Arg/ICG NCs were also investigated at the power of 1.0 W cm^{-2} for 10 min, respectively. Photothermal images were collected by an infrared thermal imaging camera.

The photothermal conversion efficacy (η) was calculated using the following equations:

$$\tau_s = \frac{m_d c_d}{hS}$$

$$Q_0 = hS(T_{max,water} - T_{surr})$$

$$\eta = \frac{hS(T_{max} - T_{surr}) - Q_0}{I(1 - 10^{-A808})}$$

In the equations, τ_s (312.02) can be calculated from the linear regression curve in the cooling curve. M_d and C_d indicate the mass of Ag_2S@ZIF-90/Arg/ICG NCs solution (1 g) and the heat capacity (4.2 J g^{-1} K^{-1}), respectively. Q_0 represents the background energy input without the photothermal agent, $T_{max,\ water}$ represents the steady-state maximum temperature of water (27.2◦C), and T_{surr} is the ambient room temperature (27 ˚C). T_{max} presents the steady maximum temperature of Ag_2S@ZIF-90/Arg/ICG NCs solution (47.8 ˚C), *I* is the laser power, and A_{808} is the absorbance of Ag_2S@ZIF-90/Arg/ICG NCs at 808 nm (0.632). Accordingly, the photothermal conversion efficiency could be calculated as 36.2%.

1.6 The pohotodynamic performance of Ag_2S@ZIF-90/Arg/ICG NCs

ROS production from different samples with NIR radiation was measured with DPBF. DPBF solution (1 mM, dissolved in ethanol) was prepared in the dark. Ag_2S@ZIF-90/Arg/ICG NCs were evenly dispersed in ethanol (0.092 mM) and then NCs were mixed into DPBF solution at a ratio of

1:1 (total volume was 1 mL). After irradiation with 808 nm NIR (1 W cm^{-2}) for different times, the supernatant was collected and the absorption spectrum of residual DPBF was determined by UV-vis spectrophotometer. ABDA was used to detect singlet oxygen. The specific method is the same as before.

Ag_2S@ZIF-90/Arg/ICG NCs was irradiated with an 808 nm NIR continuous laser at a power density of 1 W cm^{-2} for 5 min. The EPR spectra were detected on a Bruker EMXplus spectrometer operating at the X-band for identification of different species of ROS and $ONOO^-$.

1.7 Quantification of ROS

In order to correlate the fluorescent intensities and concentrations in terms of equivalent H_2O_2 concentrations, an assay for standard solutions of hydrogen peroxide was performed [5]. Six solutions of H_2O_2 concentrations of approximately 0, 250, 500, 1000, 2000 and 4000 nM were added to the 96-well plate with 50 μL each well and then 50 μL of DCFH-DA (5 μM) was added to each of the above wells. Standard solutions were incubated at 37 ˚C for 15 min. The 0.184 mM NCs (50 μL) is then mixed with 50 μL DCFH-DA solution and irradiated with 808 nm NIR (1W cm^{-2} 10min). The above solution was incubated at 37 °C for 15 min. The resulting fluorescent intensity was measured by a microplate reader (Synergy HT, BIO-TEK, Winooski, VT, USA). The excitation and emission wavelengths were 485 nm and 530 nm respectively.

1.8 Measurement of NO generation

The NO release behaviors were measured using Nitric Oxide kits. Firstly, commercial $NaNO_2$ (0, 5 μM, 10 μM, 20 μM, 40 μM and 80 μM) was used to construct standard curves (**Figure S13a**). Then, the NO production by Ag_2S@ZIF-90/Arg/ICG NCs (0, 0.046 mM, 0.092 mM, 0.184 mM) irradiated with NIR (0, 0.5 W cm^{-2}, 1.0 W cm^{-2}, 2.0 W cm^{-2}) for 15 min was measured using Griess reagent. Briefly, the Ag_2S@ZIF-90/Arg/ICG NCs were uniformly dispersed in PBS. After illumination, 50 μL of the supernatant was quickly added to Griess reagent to form a diazo compound that could be detected at 540 nm by the microplate reader.

1.9 Stability detection

Appearance, zeta potential and UV-vis spectra were used to measure the stability of Ag_2S@ZIF-90/Arg/ICG NCs at different time points (0 h, 12 h, 24 h, 72 h, and 1 w) and pH values (pH=5.5, 7.0 and 8.5). For the Ag_2S@ZIF-90/Arg/ICG NCs solutions with different pH (pH=5.5, 7.0 and 8.5), the ability of NO and $^1O_2$ production was detect by Griess assay and DPBF probe.

1.10 Bacterial Biofilm Formation

All two periodontal pathogens used in this study were obtained from American Type Culture Collection (ATCC, Manassas, VA): *P. gingivalis* (ATCC33277) and *F. nucleatum* (ATCC 10953). The bacterial species was approved by the Institutional Review Board of Jilin University School of Stomatology. The inoculum was adjusted to 1×10^8 CFU mL^{-1} in 1 mL medium into 24-well plates with round glass climbing sections. All bacteria were cultured in BHI medium at 37 °C for 96 h, and the medium was freshly replaced every 24 h.

1.11 Resistance to free pathogenic bacteria

Minimum inhibitory concentration (MIC) and minimal bacterialcidal concentration (MBC) were used to detect resistance to free pathogenic bacteria. Bacteria were cultured in BHI medium at 37 °C for 10 h, and then the bacterial suspension was diluted in BHI medium to a cell density of 2×10^5 colony forming units (CFU) mL^{-1} as the working suspension. The Ag_2S@ZIF-90/Arg/ICG NCs were serially diluted by BHI medium in a 96-well plate, and an equal volume of bacterial suspension was added into each well. The final concentration of Ag_2S@ZIF-90/Arg/ICG NCs was ranging from 100 to 6.25 μg mL^{-1}. All the wells were irradiated with 808 nm NIR (1 W cm^{-2}) for 5 min and then the plates were incubated at 37 °C for 24 h. Wells with inoculum and no NCs were used as the positive control; wells with only BHI medium were used as the blank.

The time-kill curve was used to determine the killing activity of Ag_2S@ZIF-90/Arg/ICG NCs against planktonic bacteria (*P. gingivalis* and *F. nucleatum*). Briefly, *P. gingivalis*, and *F. nucleatum* suspensions were diluted to 2×10^5 CFU mL^{-1}. The bacteria suspensions were treated with Ag_2S@ZIF-90/Arg/ICG NCs at 0.092 mM with 808 nm irradiation at an intensity of 1 W cm^{-2} for 5 min every hour. Upon 0, 1, 2, 4, 6, 8, 10 and 12 h incubation, the suspensions were 10-fold serially-diluted and spread onto Columbia blood agar. After incubation for 48 h at 37 °C, the CFU was counted.

1.12 ROS staining in biofilm

ROS in biofilm was stained by a commercial ROS indicator 2,7-dichlorofluorescein diacetate (DCFH-DA). After Ag_2S@ZIF-90/Arg/ICG NCs and biofilm were co-incubated at 37 °C for 72 h, they were treated with 808 nm NIR irradiation (1 W cm^{-2} 5min). Finally, the biofilm was treated with the DCFH-DA solution (5 μM) at 37 °C for 30 min.

1.13 Quantitative detection of ROS produced by Ag_2S@ZIF-90/Arg/ICG NCs in biofilms

The 100 μL BHI medium containing 0.184 mM Ag_2S@ZIF-90/Arg/ICG NCs was mixed with biofilm to co-culture in 96-well plates for 3 days. 100 μL DCFH-DA (5 μM) solution was added into the above wells and irradiated with 808 nm NIR (1W cm^{-2} 10 min). In order to exclude the influence of biofilm and BHI medium, the wells with biofilm were set as the control. The above

96-well plate was incubated at 37 °C for 15 min. The resulting fluorescent intensity was measured by the microplate reader. The excitation and emission wavelengths were 485 nm and 530 nm respectively. The final fluorescence intensity was: FI (0.092 mM Ag_2S@ZIF-90/Arg/ICG NCs)－FI (control). Finally, the ROS output was calculated by using the standard curve in the **Figure S10B**.

1.14 Standard plate count

After incubation, the samples were collected into 1 mL sterile PBS and ultrasonic to dislodge the adhered bacteria on surface of samples. The obtained *P. gingivalis* and *F. nucleatum* suspension were serially diluted and used for standard plate counting tests. 10 μL of the dilution suspension was dripped into Columbia blood agar and incubated at 37 °C for 96 h. The CFUs were imaged and counted.

1.15 Live/dead staining of biofilms

In addition, a mixture of SYTO9 and PI dyes was used for fluorescent-based biofilm live/dead observations. The glass samples with biofilm were stained with dye for 40 min. The images were observed via confocal laser scanning microscope.

1.16 Metabolic activity of biofilms

The 3-(4,5-dimethylthiazol-2-yl)-2,5-diphenyltetrazolium bromide (MTT) assay was used to investigate the metabolic activity of the biofilms. The round glass discs with biofilms were treated with MTT in a new 24-well plate and incubated at 37 °C in 5% CO_2 for 1 h. Equal volume of DMSO was added to dissolve Formazan crystals. The plate was shaken horizontally for 20 min in the dark. The mixed solution (100 μL) was added into a new 96-well plate. Absorbance at OD 540 nm was measured by a microplate reader (BioTek).

1.17 SEM images of P. gingivalis and F. nucleatum

For biofilm SEM images, *P. gingivalis* and *F. nucleatum* biofilms were respectively cultured with various NCs at 24-well plate for 72 h. Then wells with biofilm were irradiated with 808 nm NIR (1 W cm^{-2} 5 min) for 3 days. Afterwards, the biofilm was immobilized using 2.5% glutaralde-hyde for 12 h. Then, the bacterium was dehydrated with different proportions of ethanol solution. Finally, the samples were observed using SEM.

1.18 NIR-II fluorescence imaging of the animal models

The following experiments were based on previous literature with slight adjustments [6]. Animals imaging of NIR-II fluorescence was implemented with NIR imaging system (Teledyne Princeton Instruments, California, USA). 200 μL Ag_2S@ZIF-90/Arg/ICG NCs solution (0.092 mM) and ICG solution (2.65 μg mL^{-1}) were separately injected into subgingival of the anterior teeth of Wistar rats. Meanwhile, the above two solutions were respectively pipetted into the mouth of Wistar rats, and then the rats were euthanized to extract mandibles. Rats in the local injection groups and the oral administration groups were exposed to the excitation laser (808 nm 50 mW cm^{-2} 1-2 s) to

get NIR-II fluorescence imaging.

1.19 Immunofluorescence staining for activation inhibition of NF-κB pathway

Firstly, biofilms with a *P. gingivalis* and *F. nucleatum* (1:1 with 10^8 CFU mL^{-1}) were cultured in 24-well plates. After biofilm matured, BHI medium and biofilm were thoroughly mixed to form suspensions for future use. Mouse macrophage RAW 264.7 was used to establish inflammation model *in vitro*, and Ag_2S@ZIF-90/Arg/ICG NCs inhibited NF-κB pathway was evaluated. 2×10^5 RAW 264.7 cells/well were inoculated in 6-well plates for 24 h. Then, 2 mL culture mediums containing 200 μL biofilm suspensions were added in different wells as inflammatory stimulation to simulate the stimulation of macrophages by c-di-AMP in biofilm. The blank control group was refresh with normal culture medium. The inflammatory group and other experiment groups were replaced with culture medium containing 200 μL biofilm suspensions. After 6 h, they were replaced with 2 mL normal mediums or culture mediums containing different NCs for treatment. After fixing with 4% paraformaldehyde, the cells were incubated with anti-NF-κB/p65 primary antibodies (1:500), followed by incubation with IgG secondary antibodies (1:1000) and DAPI. The NF-κB/p65-positive cells were identified and counted under a microscope.

1.20 In vitro cytotoxicity assay

To examine the cellular dark toxicity of Ag_2S@ZIF-90/Arg/ICG NCs, the relative survival rate of mouse fibroblast L929 cells was detected by the CCK-8 method. Cells were seeded in 96-well plates at a density of 5000 cells/well and cultured for 24 h. Subsequently, the different concentrations of Ag_2S@ZIF-90/Arg/ICG NCs (0 mM, 0.046 mM, 0.092 mM, 0.184 mM) were added to mouse fibroblast L929 cells, and then the cells were incubated for 24 and 72 h in the dark to evaluate the cytotoxicity of Ag_2S@ZIF-90/Arg/ICG NCs.

In addition, an xCELLigence real-time cell analysis (RTCA) Instrument was used to constantly monitor the cell viability by Ag_2S@ZIF-90/Arg/ICG NCs at different concentrations (0 mM, 0.046 mM, 0.092 mM, 0.184 mM). Briefly, cells were seeded at a density of 3×10^3 cells/well in an E-plate and incubated for 24 h. Then, different concentrations of Ag_2S@ZIF-90/Arg/ICG NCs medium were changed.

The human gingival fibroblasts (HGFs) morphology was observed via fluorescein isothiocyanate (FITC, Sigma-Aldrich) and 4', 6-diamidino-2-phenylindole (DAPI, Sigma-Aldrich) staining. HGFs were incubated in a 24-well plate at 37 °C for 24 h. Then the cells were treated with different concentrations of Ag_2S@ZIF-90/Arg/ICG NCs for another 24 h. Cells without

nanocomposites were used as control. the cells were fixed with 4% paraformaldehyde. Then the cells were stained with FITC/DAPI following the manufacturer's instructions. The cell morphology was detected by CLSM. All measurements were tested in three replicates.

1.21 Hemolysis assay

The plasma and red blood cells (RBC) were obtained from fresh rabbit blood. The RBC was re-suspended in PBS and added with different nanoparticles at 37 °C for 2 h. Hemoglobin release was analyzed by a microplate analyzer at 576 nm, and the triton-X 100 and PBS were used as positive and negative controls, respectively. Six samples were tested in each group [7]. The following formula was used to calculate hemolysis percentage:

Hemolysis ratio (%) = (OD sample－OD negative)/(OD positive－OD negative) × 100%.

1.22 The toxicity in vivo.

Wistar rats (six-week-old, male) were used under protocols approved by the Committee for Animal Experimental Ethical Inspection of Jilin University (#SY2022-51), China. Different nanocomposites were injected into the gingival tissue of the mandibular incisor. Then the Wistar rats were sacrificed after 10 weeks of normal feeding.

To evaluate the biosafety of Ag_2S@ZIF-90/Arg/ICG NCs *in vivo*, the major organs of heart, liver, spleen, lung, and kidney of rats were collected, infiltrated with xylene and embedded with paraffin to perform the H&E staining.

2. Supplementary Tables

Table S1. Primer sequences used in this study

Sample	Ag_2S NPs	Ag_2S@ZIF-90 NCs	Ag_2S@ZIF-90/Arg NCs	Ag_2S@ZIF-90/Arg/ICG NCs
TEM	35 nm	50-60 nm	50-60 nm	50-60 nm
DLS	52 nm	102 nm	104 nm	105 nm

Table S2. N_2 adsorption/desorption data of Ag_2S@ZIF-90 and its derivatives.

Sample	BET surface area ($m^2\ g^{-1}$)	Langmuir surface area ($m^2\ g^{-1}$)	Pore volume ($cm^3\ g^{-1}$)
Ag_2S@ZIF-90	823.753 $m^2\ g^{-1}$	1036.510 $m^2\ g^{-1}$	0.659 $cm^3\ g^{-1}$
Ag_2S@ZIF-90/Arg	203.933 $m^2\ g^{-1}$	271.587 $m^2\ g^{-1}$	0.602 $cm^3\ g^{-1}$
Ag_2S@ZIF-90/Arg/ICG	33.561 $m^2\ g^{-1}$	41.037 $m^2\ g^{-1}$	0.478 $cm^3\ g^{-1}$

Table S3. Primer sequences used in the antibacterial experiment

Gene	Forward Sequence (5' to 3')	Reverse Sequence (5' to 3')
16s rRNA	TGTAGATGACTGATGGTGAAA	ACTGTTAGCAACTACCGATGT
fimA	CAGCAGGAAGCCATCAAATC	CAGTCAGTTCAGTTGTCAAT
hagA	ACAGCATCAGCCGATATTCC	CGAATTCATTGCCACCTTCT
hagB	TGTCGCACGGCAAATATCGCTAAAC	CTGGCTGTCCTCGTCGAAAGCATAC
Kgp	AGCTGACAAAGGTGGAGACCAAAGG	TGTGGCATGAGTTTTTCGGAACCGT
PPAD	GGTCGCAAAGAGACTTATCCCT	TTGAACCATGCACGAAGAGC
RgpA	CTGCGAGCGGTATTAGTGGT	CTACCAGCCCGTTTCCAACT
RgpB	TCGGGACAAGTGTACGAACG	AACCAGTCTTGGGCTTCTCC
pgn1187	GGTAGAGGAGGCGGAGCGTATC	GGGCTGACGAACTCTCCAATGC
pgn2003	CGCCAACTTAATAAACGCCCTTGTC	CCACTATGCCGATAACGCCCTTG
pgn0523	CTCCGCTTATGCTCGCCTGTATG	GTTCGTCCGTGGTGATATCCTGATG
pgn0521	CGGACTGAACCTGACCAACTACTTG	CACCAGAATGAGCGAAAGCACAAAG

Table S4. Primer sequences used in this study

Gene	Forward Sequence (5' to 3')	Reverse Sequence (5' to 3')
β-actin	CATCCGTAAAGACCTCTAGCCAAC	ATGGAGCCACCGATCCACA
IL-1β	TCCAGGATGAGGACATGAGCAC	GAACGTCACACACCAGCAGGTTA
Arg-1	CAAGACAGGGCTACTTTCAGGAC	GATTACCTTCCCGTTTCGTTCC

Table S5 Quantitative analysis of ROS produced by Ag_2S@ZIF-90/Arg/ICG NCs in biofilms

Biofilm	Fluorescence intensity (a.u.) control	Fluorescence intensity (a.u.) 0.092 mM Ag_2S@ZIF-90/Arg/ICG NCs	ROS (nM)
P. gingivalis	43	286	693.41
F. nucleatum	46	291	700.13

3. Supplementary Figures

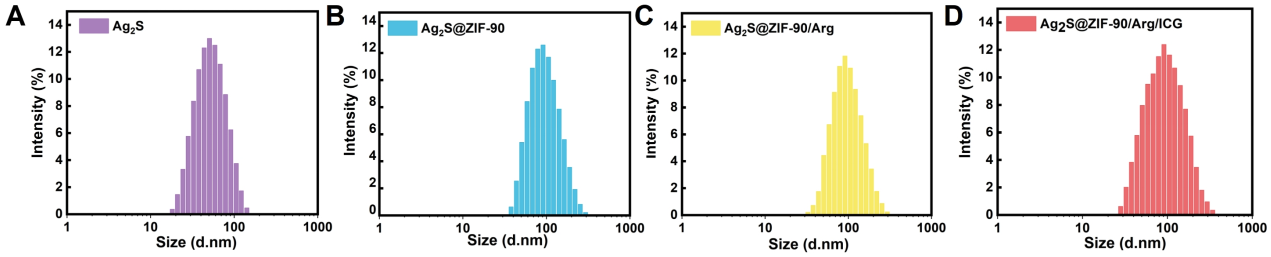

Figure S1. DLS of various NCs.

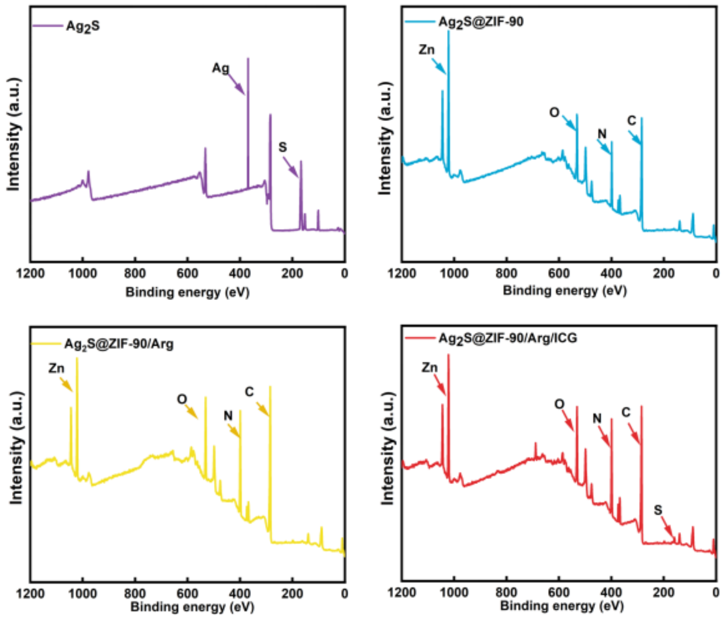

Figure S2. XPS survey spectra of Ag_2S NPs (purple), Ag_2S@ZIF-90 NCs(blue), Ag_2S@ZIF-90/Arg NCs (yellow), Ag_2S@ZIF-90/Arg/ICG NCs (red).

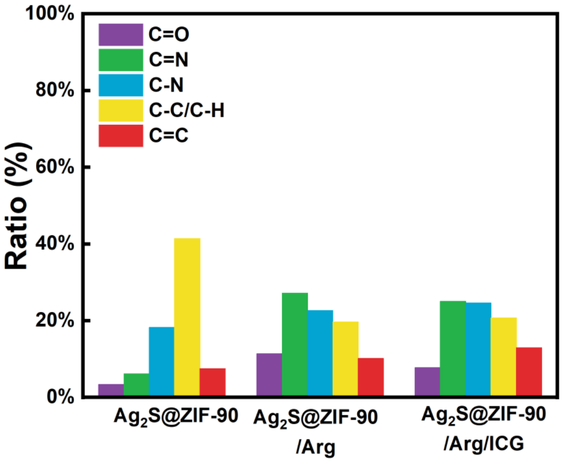

Figure S3. The proportional distribution based on C 1s spectra.

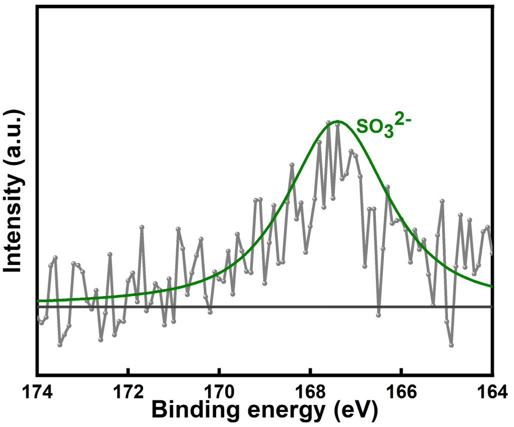

Figure S4. XPS spectra of S 2p obtained Ag_2S@ZIF-90/Arg/ICG NCs.

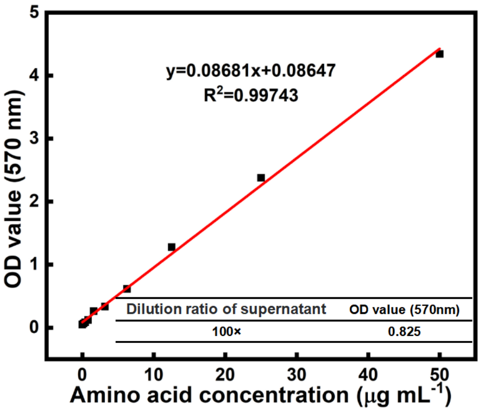

Figure S5. Standard curve of amino acid standard samples (0-50 μg mL^{-1}). (The bottom right table showed the value of ninhydrin at OD value 570 nm generated by amino acids after 100 dilutions of the supernatant. The weights of L-arg loading and input were approximately 25.52 mg and 34.42 mg.)

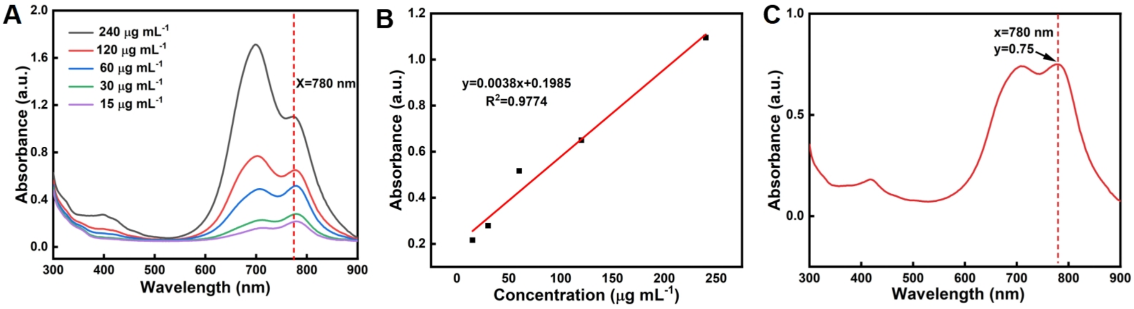

Figure S6. (A) UV-vis spectrum of free ICG at different concentrations. (B) Standard curve of free ICG absorbance at specific absorbing peak at 780 nm. (C) UV-vis spectrum of first centrifugal fluid of Ag_2S@ZIF-90/Arg/ICG.

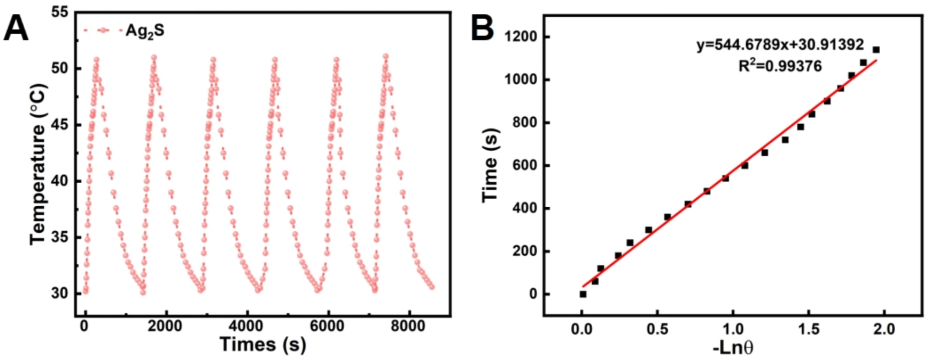

Figure S7. (A) Temperature elevations of Ag_2S NPs for six 808 nm NIR irradiations cycles. (B) Curve of cooling time versus negative natural logarithm of the driving force temperature obtained from a cooling stage to calculate time constant (τs) for heat transfer.

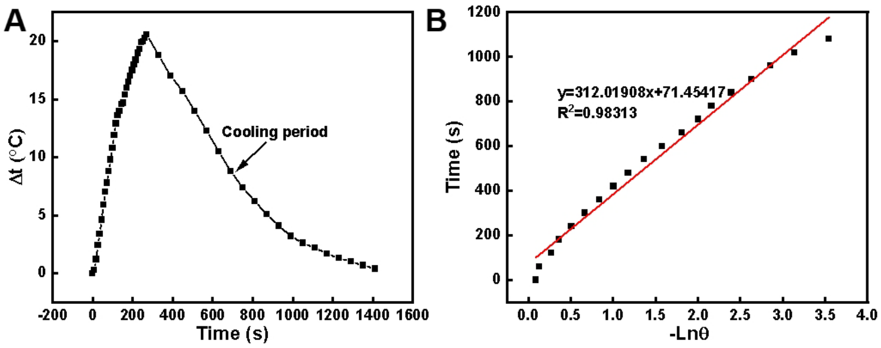

Figure S8. (A) Temperature elevations of Ag_2S@ZIF-90/Arg/ICG NCs for 808 nm NIR irradiation cycle. (B) Curve of cooling time versus negative natural logarithm of the driving force temperature obtained from a cooling stage to calculate time constant (τ_s) for heat transfer.

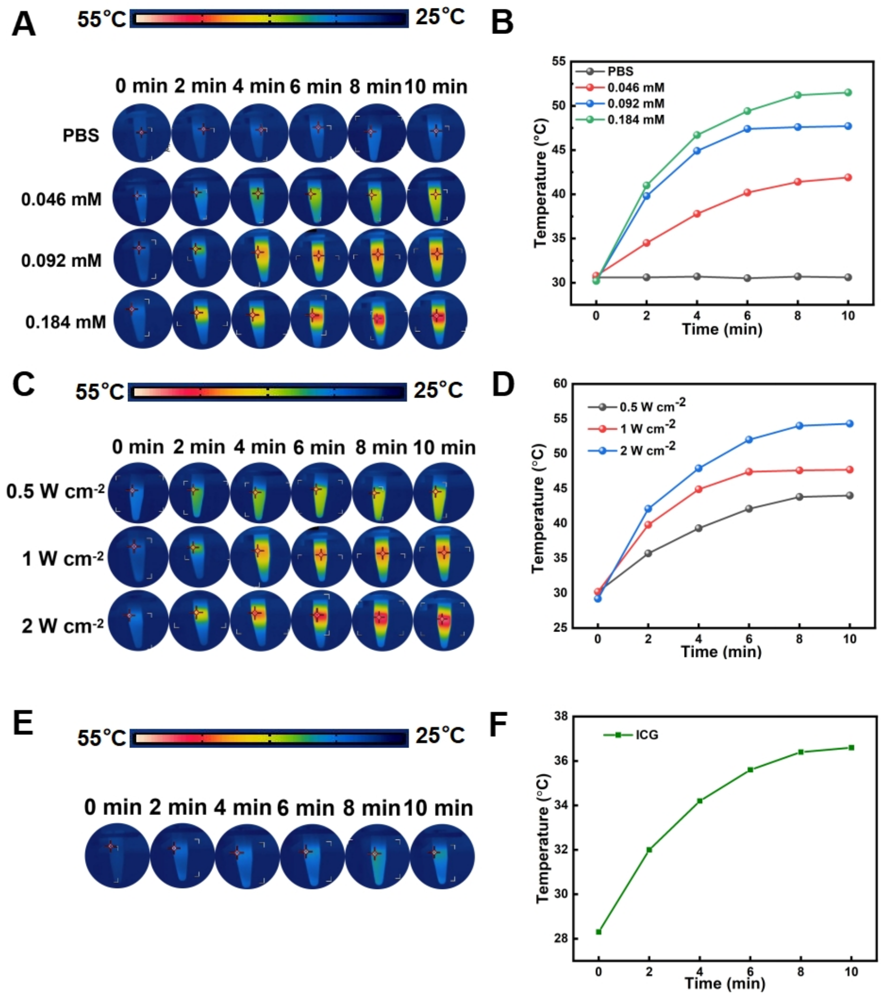

Figure S9. (A) Infrared thermal images of Ag_2S@ZIF-90/Arg/ICG NCs at different concentrations (0.046 mM, 0.092 mM, 0.184 mM) and (B) corresponding temperature profiles. (C) different light intensities under an 808 nm NIR (0.5 W cm^{-2}, 1 W cm^{-2}, 2 W cm^{-2}, 10 min) and (D) corresponding temperature profiles. (E) Infrared thermal images of free ICG at the upload equivalent yield in 0.092 mM Ag_2S@ZIF-90/Arg/ICG NCs under an 808 nm NIR (1 W cm^{-2}, 10 min) and (F) corresponding temperature profiles of free ICG.

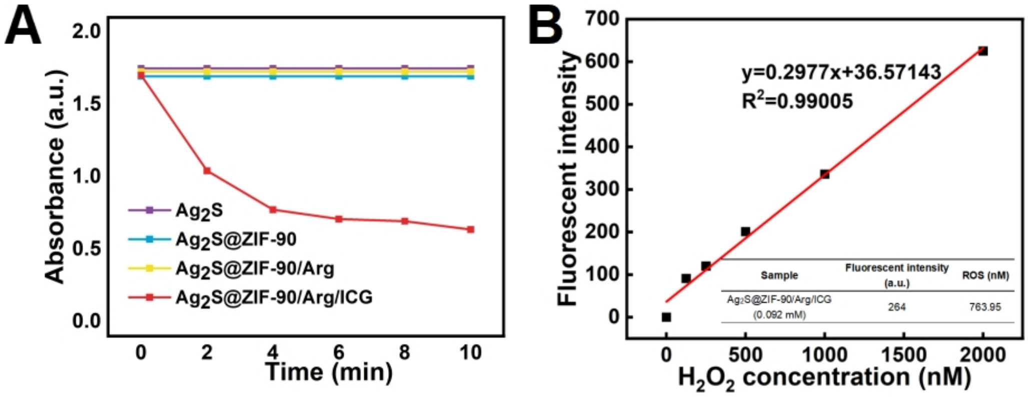

Figure S10. (A) Depletion of DPBF (OD value at 410 nm) for different nanocomposites. (B) Standard curve of fluorescence intensity of DCF produced by oxidation of DCFH-DA by H_2O_2 standard substance (The fluorescence intensity of DCF produced by ROS oxidation of DCFH-DA and the amount of ROS produced by Ag_2S@ZIF-90/Arg/ICG NCs was shown in the lower right corner)

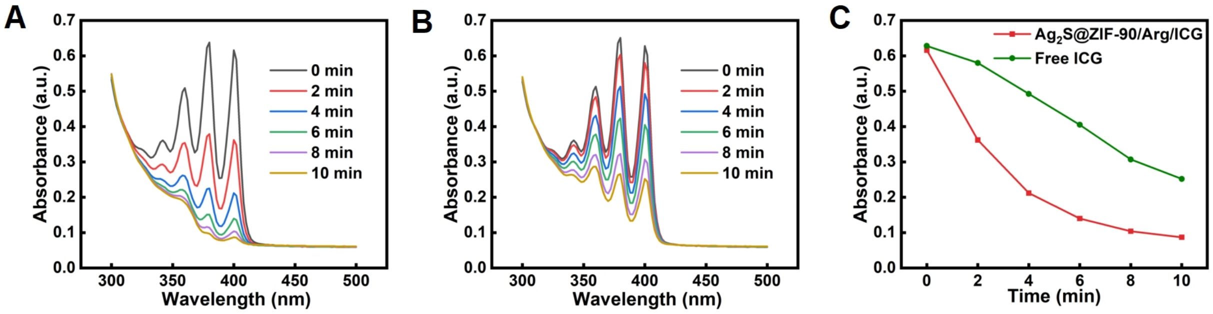

Figure S11. (A-B) The decomposition of ABDA in the presence of (A) Ag_2S@ZIF-90/Arg/ICG NCs and (B) free ICG under 808 nm NIR irradiation. (C) Changes of OD value at 400 nm after Ag_2S@ZIF-90/Arg/ICG NCs and ICG consumed ABDA after 808 nm NIR laser irradiation.

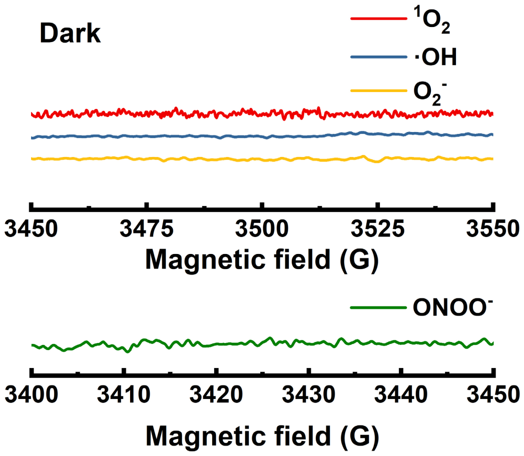

Figure S12. EPR spectrum of reactive radicals $^1O_2$, ·OH, O_2^- and $ONOO^-$ without 808 nm NIR irradiation.

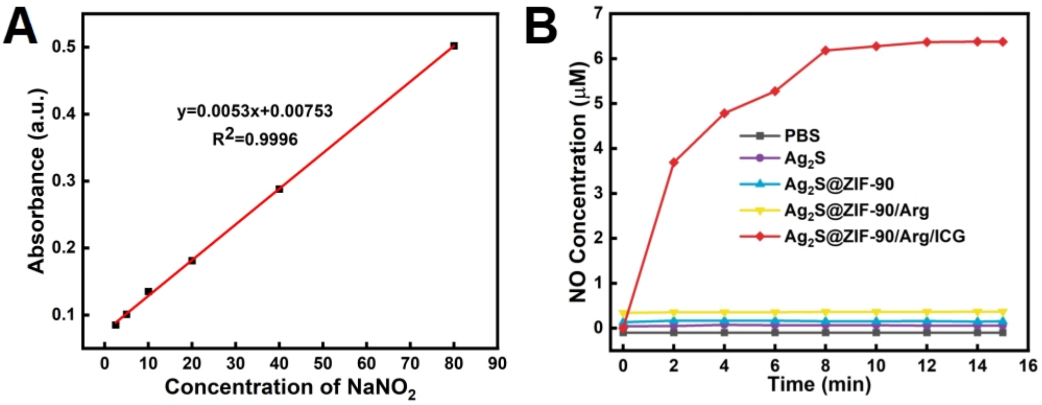

Figure S13. (A) Standard curve of $NaNO_2$ standard samples (0-80 μM). (B) NO production concentrations of different nanocomposites.

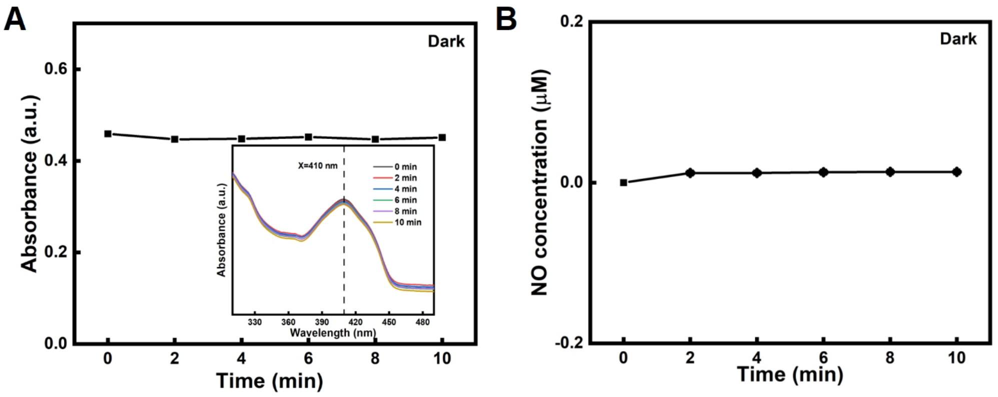

Figure S14. (A) Depletion of DPBF (OD value at 410 nm) for Ag_2S@ZIF-90/Arg/ICG NCs under dark condition. (B) NO concentration of Ag_2S@ZIF-90/Arg/ICG NCs under dark condition.

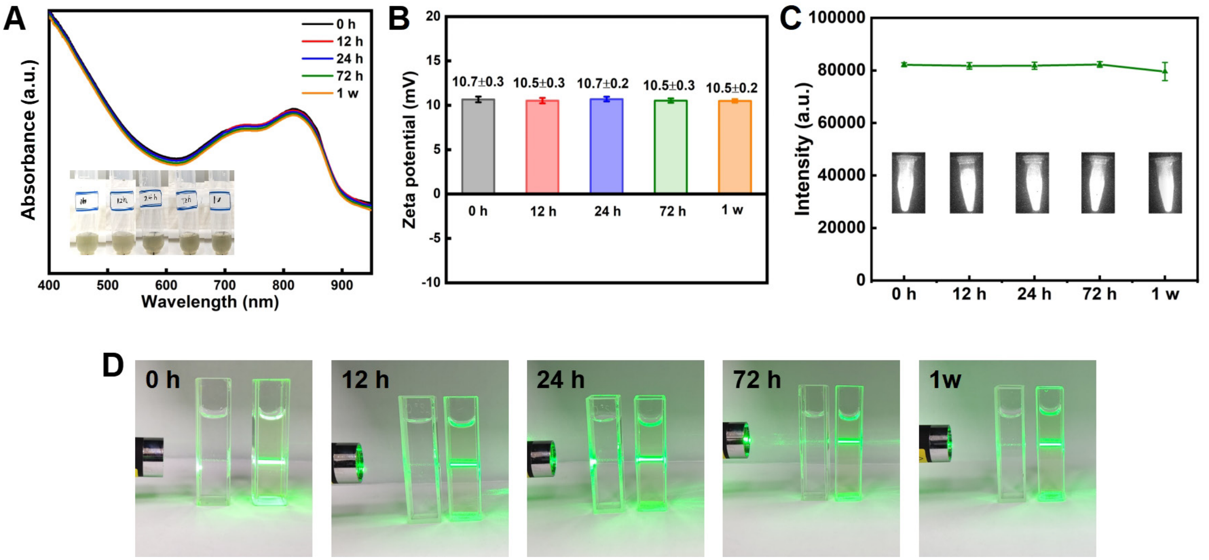

Figure S15. (A) UV-vis spectra of Ag_2S@ZIF-90/Arg/ICG NCs at different time points. (B) Zeta potential of Ag_2S@ZIF-90/Arg/ICG NCs at different time points. ("h" means hour; "w" means week). (C) NIR-II fluorescent intensity and images of Ag_2S@ZIF-90/Arg/ICG NCs at different time points. (D) Tyndall effect of Ag_2S@ZIF-90/Arg/ICG NCs at different time points (The left cuvette contained deionized water and the right cuvette contained Ag_2S@ZIF-90/Arg/ICG NCs.)

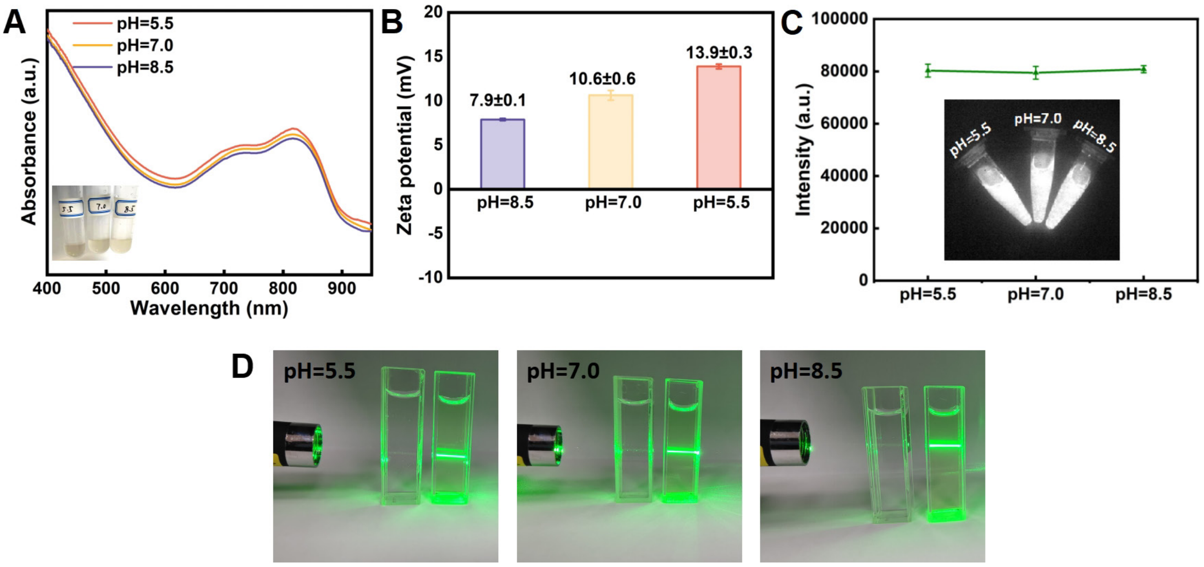

Figure S16. (a) UV-vis spectra of Ag_2S@ZIF-90/Arg/ICG NCs at different pH values and images of Ag_2S@ZIF-90/Arg/ICG NCs (left bottom) at different pH values. (b) Zeta potential of Ag_2S@ZIF-90/Arg/ICG NCs at different pH values. (C) NIR-II fluorescent intensity and images of Ag_2S@ZIF-90/Arg/ICG NCs at different pH values. (D) Tyndall effect of Ag_2S@ZIF-90/Arg/ICG NCs at different pH values. (The left cuvette contained deionized water and the right cuvette contained Ag_2S@ZIF-90/Arg/ICG NCs.)

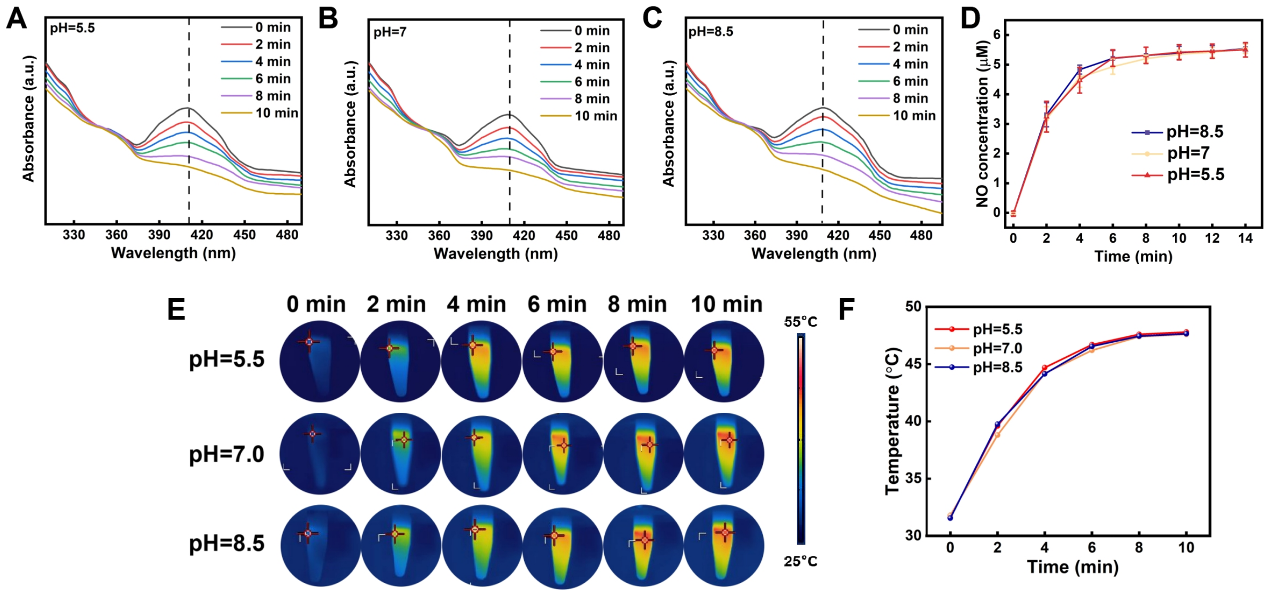

Figure S17. (A-C) Depletion of DPBF for Ag_2S@ZIF-90/Arg/ICG NCs at different pH values of (A) 5.5, (B) 7.0, and (C) 8.5. (D) NO concentration of Ag_2S@ZIF-90/Arg/ICG NCs at different pH values. (E) Infrared thermal images of Ag_2S@ZIF-90/Arg/ICG NCs at different pH values and (F) corresponding temperature profiles.

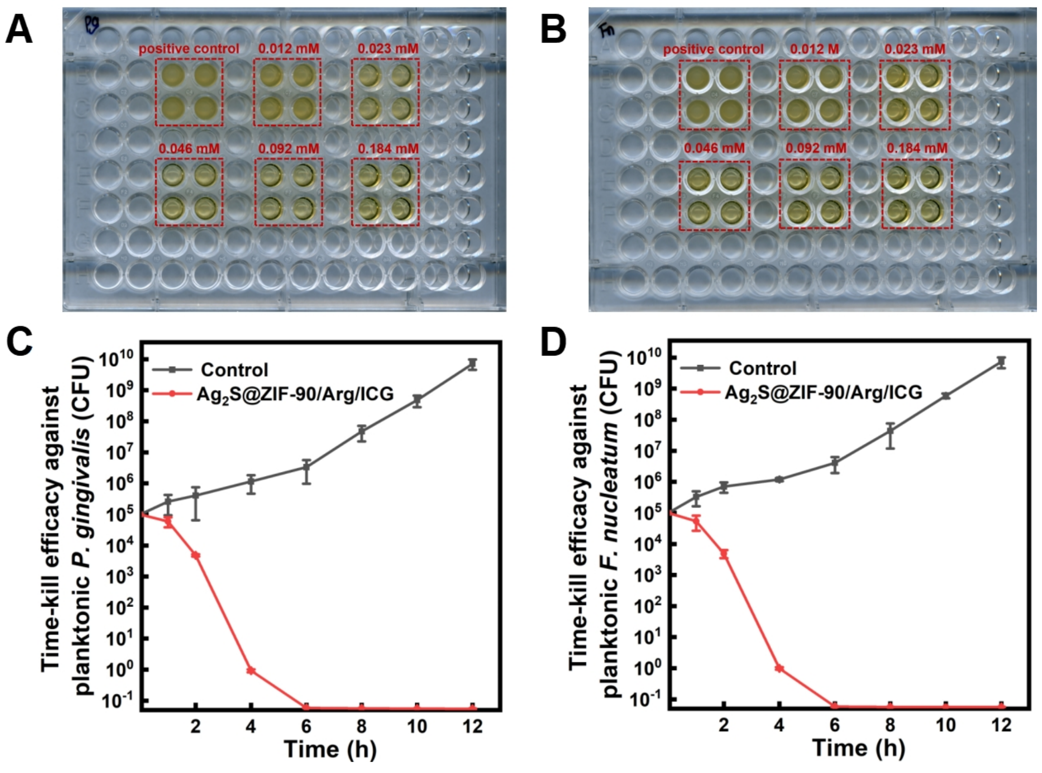

Figure S18. (A-B) Images of 96-well plates from the MIC and MBC experiments of (A) *P. gingivalis* and (B) *F. nucleatum*. (C-D) Time-kill curve of Ag_2S@ZIF-90/Arg/ICG NCs in the presence of 808 nm NIR irradiation against (C) *P. gingivalis* and (D) *F. nucleatum*.

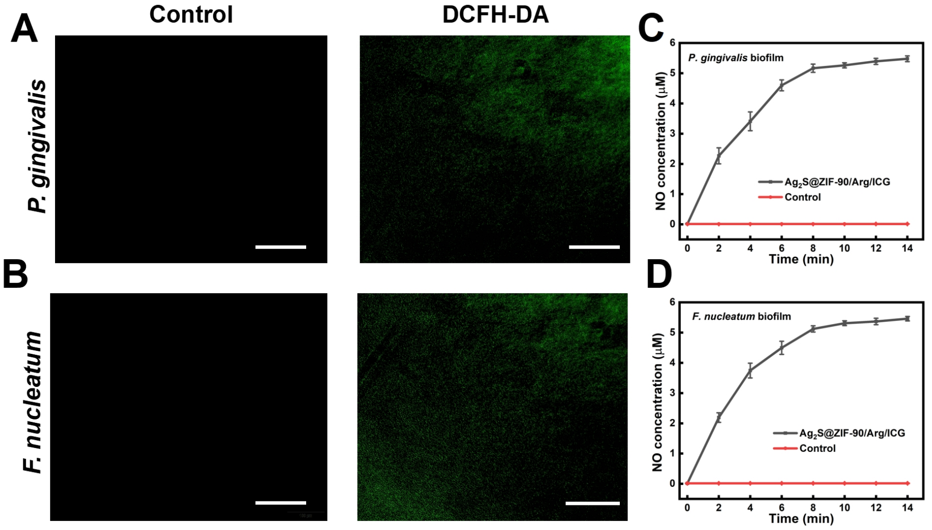

Figure S19. The ability of Ag_2S@ZIF-90/Arg/ICG NCs to produce ROS and NO in biofilm. (A-B) ROS produced by Ag_2S@ZIF-90/Arg/ICG NCs are captured by DCFH-DA fluorescent probes in (A) *P. gingivalis* biofilm and (B) *F. nucleatum* biofilm under 808 nm NIR irradiation (Scale 100 μm). (C-D) NO produced by Ag_2S@ZIF-90/Arg/ICG NCs in (C) *P. gingivalis* biofilm and (D) *F. nucleatum* biofilm under 808 nm NIR irradiation.

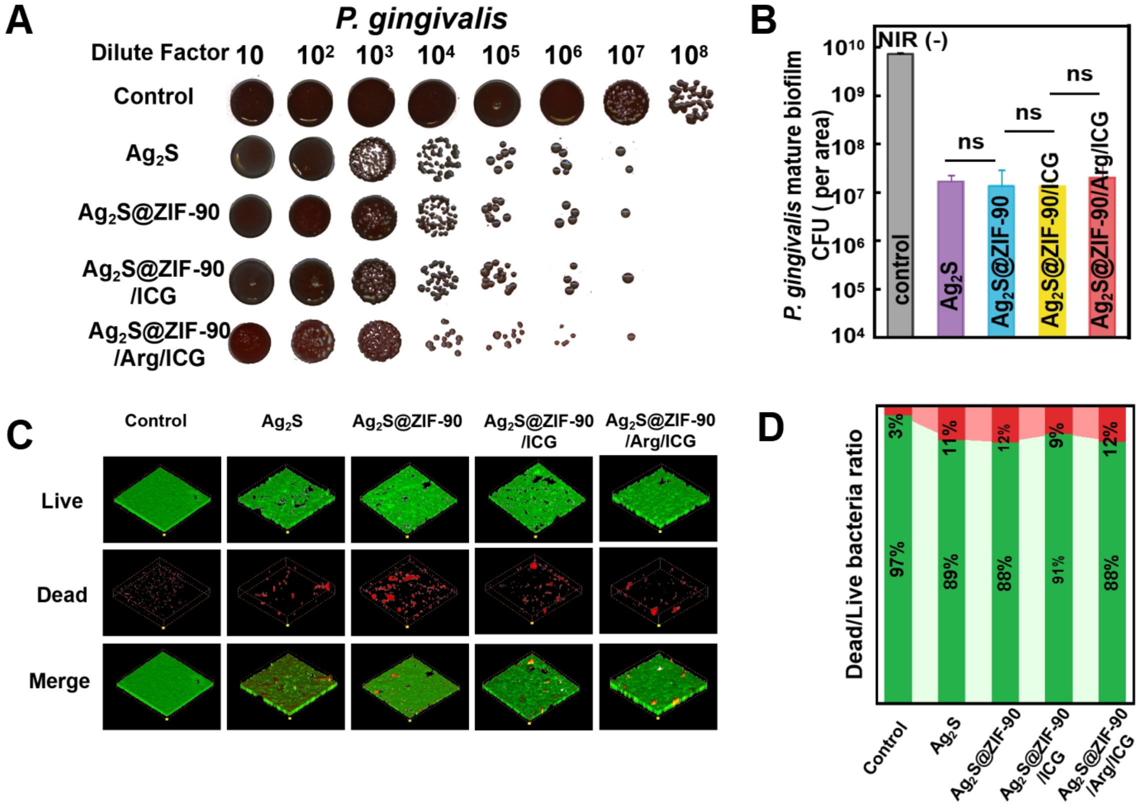

Figure S20. The capacity of various nanocomposites to resist *P. gingivalis* biofilm without 808 nm NIR irradiation. (A) Images of *P. gingivalis* clones without 808 nm NIR irradiation and (B) corresponding statistical data of colonies of *P. gingivalis*. (C) Biofilms (dead bacteria, stained red; live bacteria, stained green) and (D) corresponding Dead/Live bacteria ratio for *P. gingivalis*. Dissimilar letters indicated significant differences from each group. (ns, not significant.)

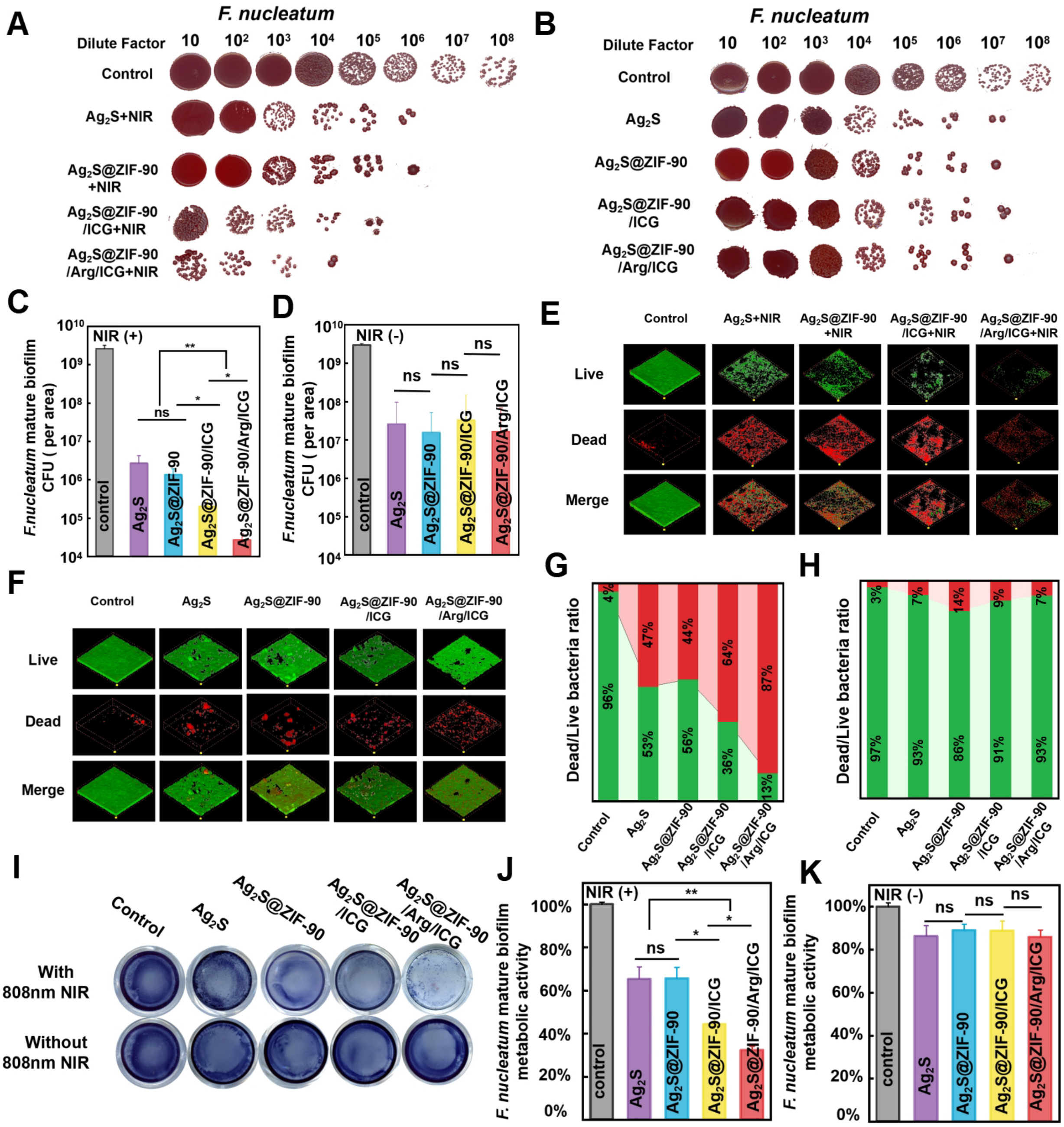

Figure S21. The capacity of various nanocomposites to resist *F. nucleatum* biofilm with and without 808 nm NIR. (A) Images of *F. nucleatum* clones with 808 nm NIR and (B) Images of *F. nucleatum* clones without 808 nm NIR. (C)(D) corresponding statistical data of colonies of *F. nucleatum*. (E) Dead and live *F. nucleatum* staining in mature biofilms with various NCs treatment with 808 nm NIR and (F) without 808 nm NIR (dead bacteria, stained red; live bacteria, stained green). (G)(H) corresponding Dead/Live bacteria ratio for *F. nucleatum*. (I) Photographs and test results of the metabolic activity of *F. nucleatum* with and without 808 nm NIR by MTT assay and (J)(K) corresponding statistical data of metabolic activity of *F. nucleatum*. (n = 3, *p < 0.05, **p < 0.01, ***p < 0.001, ns, not significant.)

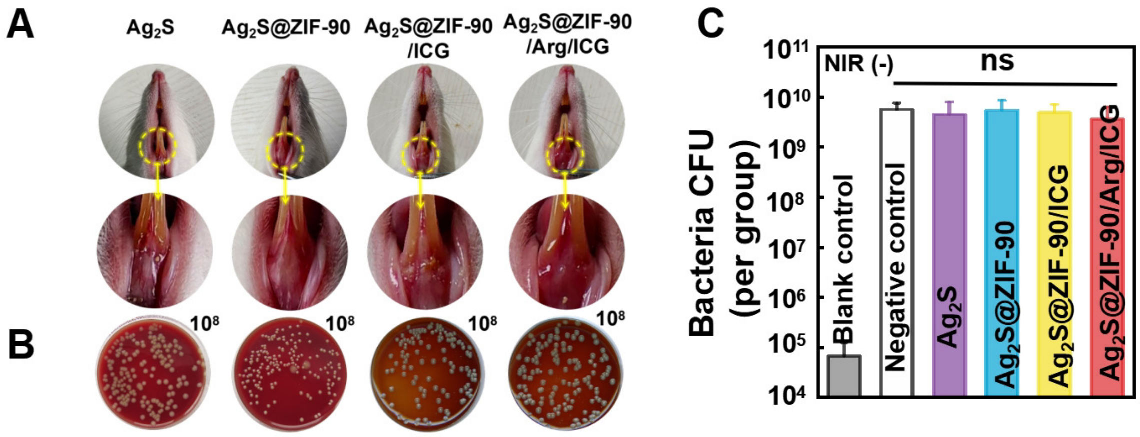

Figure S22. ***In vivo* anti-biofilm effect of different NCs without 808 nm NIR.** (A) Intraoral photos. (B) Colonies isolated from gingival tissue represent the antimicrobial function of different NCs in rats *in vivo*, the dilution factors were set in the upper right portion of the blood agar plate and (C) Corresponding bar graph of colonies. (n = 3, ns, not significant.)

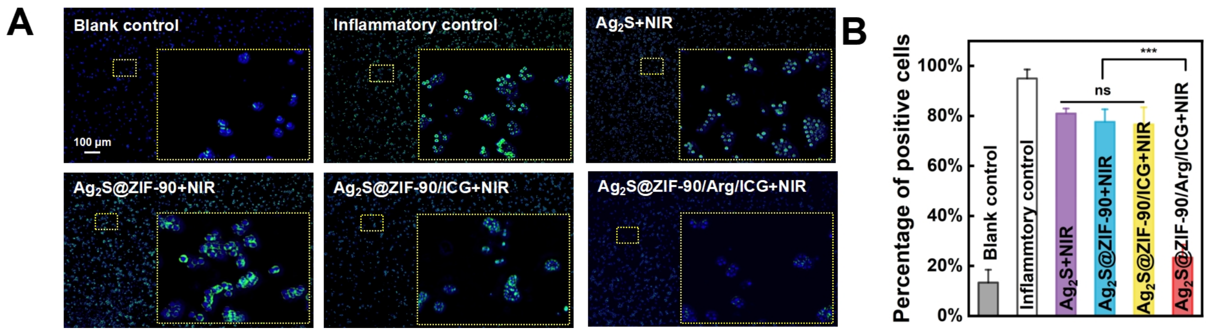

Figure S23. (A) NF-κB/p65 translocation in Raw 264.7 cells were detected by immunofluorescence analysis (The green fluorescence represents NF-κB/p65; the blue fluorescence represents DPAI) and (B) Percentage of NF-κB/p65 positive cells counted under fluorescence microscope (n = 3, ***p < 0.001, ns, not significant.)

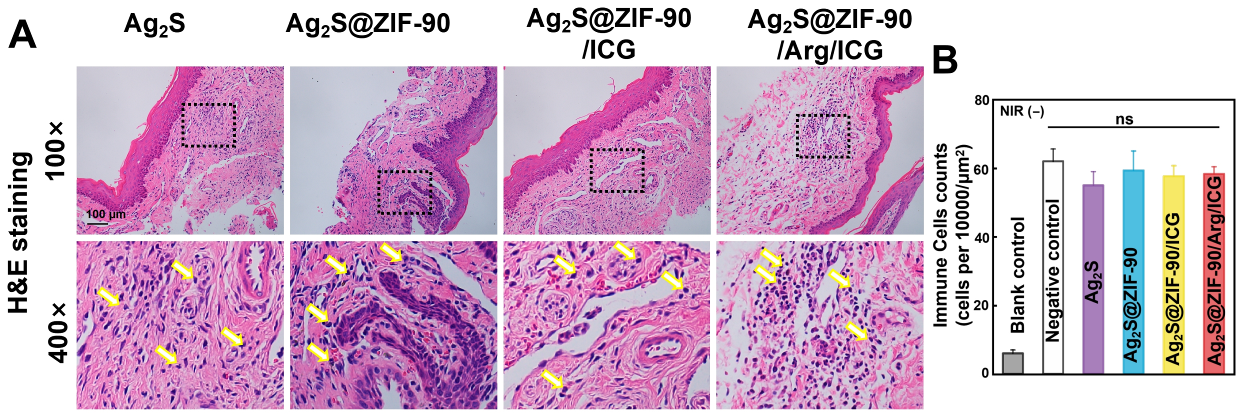

Figure S24. ***In vivo* histomorphological evaluation of inflammation of the periodontal tissue after various NCs treatment without 808 nm NIR irradiation.** (A) Typical H&E staining images (Yellow arrows: inflammation cells) and (B) Statistics of the number of immune cells at the inflammatory site of gingival tissue. (n = 3, ns, not significant.)

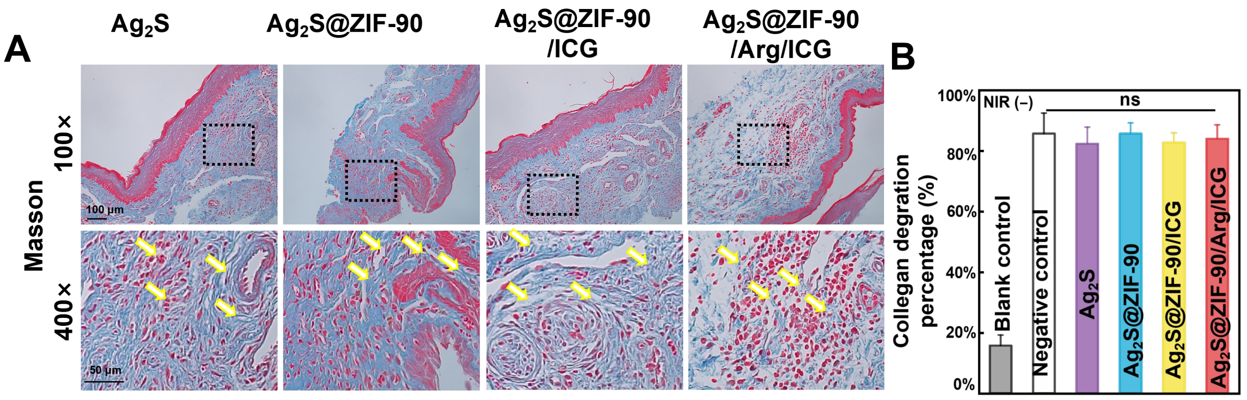

Figure S25. ***In vivo*** **histomorphological evaluation of inflammation of the periodontal tissue after various NCs treatment without 808 nm NIR irradiation.** (A) Typical Masson's staining images (Yellow arrows: Degraded collagen) and (B) Percentage of collagen degradation at the inflammatory site of gingival tissue.

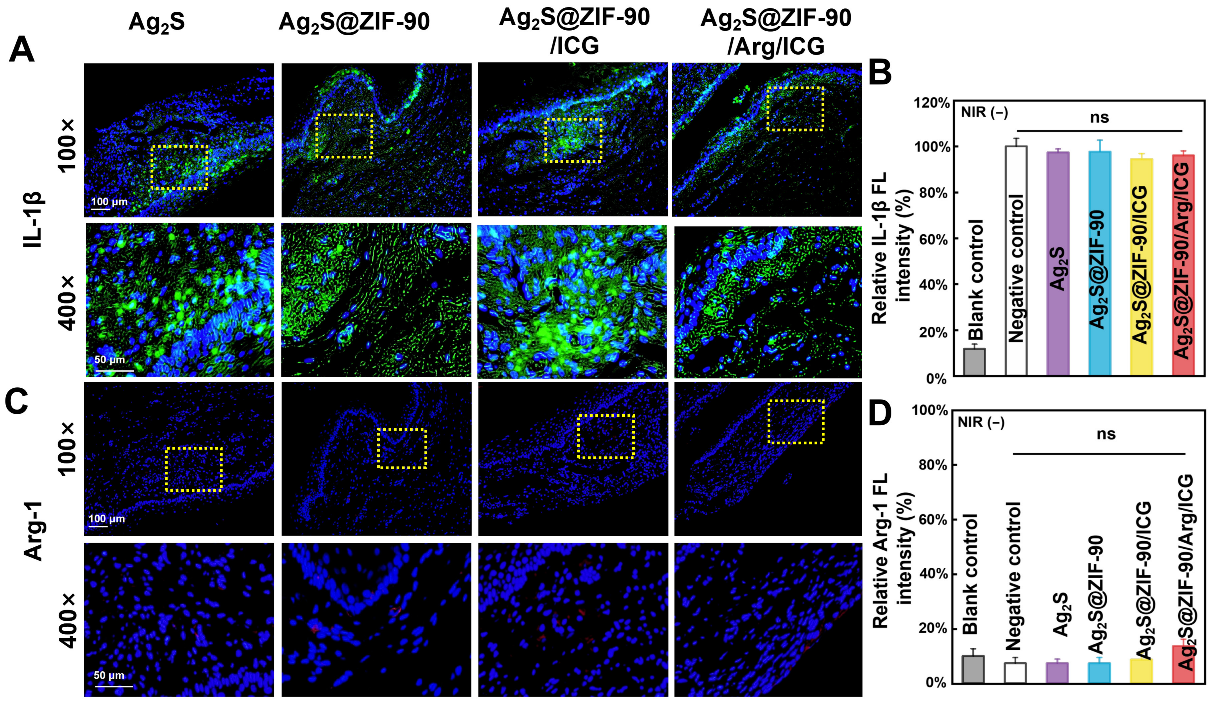

Figure S26. ***In vivo*** **histomorphological evaluation of inflammation of the periodontal tissue after various NCs treatment without 808 nm NIR irradiation.** (A) Immunofluorescence images of gingival tissue (Green fluorescence represents IL-1β positive cells and blue fluorescence represents nuclei). and (B)The corresponding immunofluorescence intensity of IL-1β. (C) Immunofluorescence images of gingival tissue (Red fluorescence represents Arg-1 positive cells and blue fluorescence represents nuclei). (D) The corresponding immunofluorescence intensity of Arg-1. (n = 3, ns, not significant.)

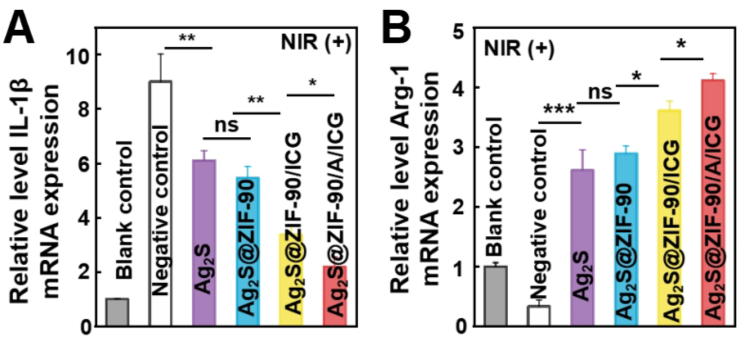

Figure S27.The RT-qPCR of gingival tissue from rat treated by various nanocomposites under 808 nm NIR irradiation. The mRNA expression relative levels of IL-1β (A) and (B) Arg-1. ($n = 3$, $^{*}p < 0.05$, $^{**}p < 0.01$,$^{***}p < 0.001$, ns, not significant.)

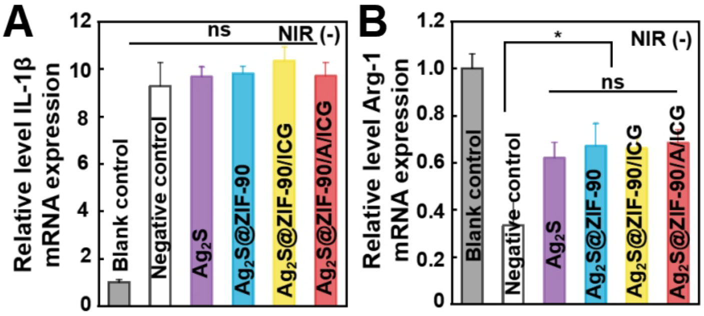

Figure S28. The RT-qPCR of gingival tissue from rat treated by various nanocomposites without 808 nm NIR irradiation. The mRNA expression relative levels of IL-1β (A) and (B) Arg-1. ($n = 3$, ns, not significant.)

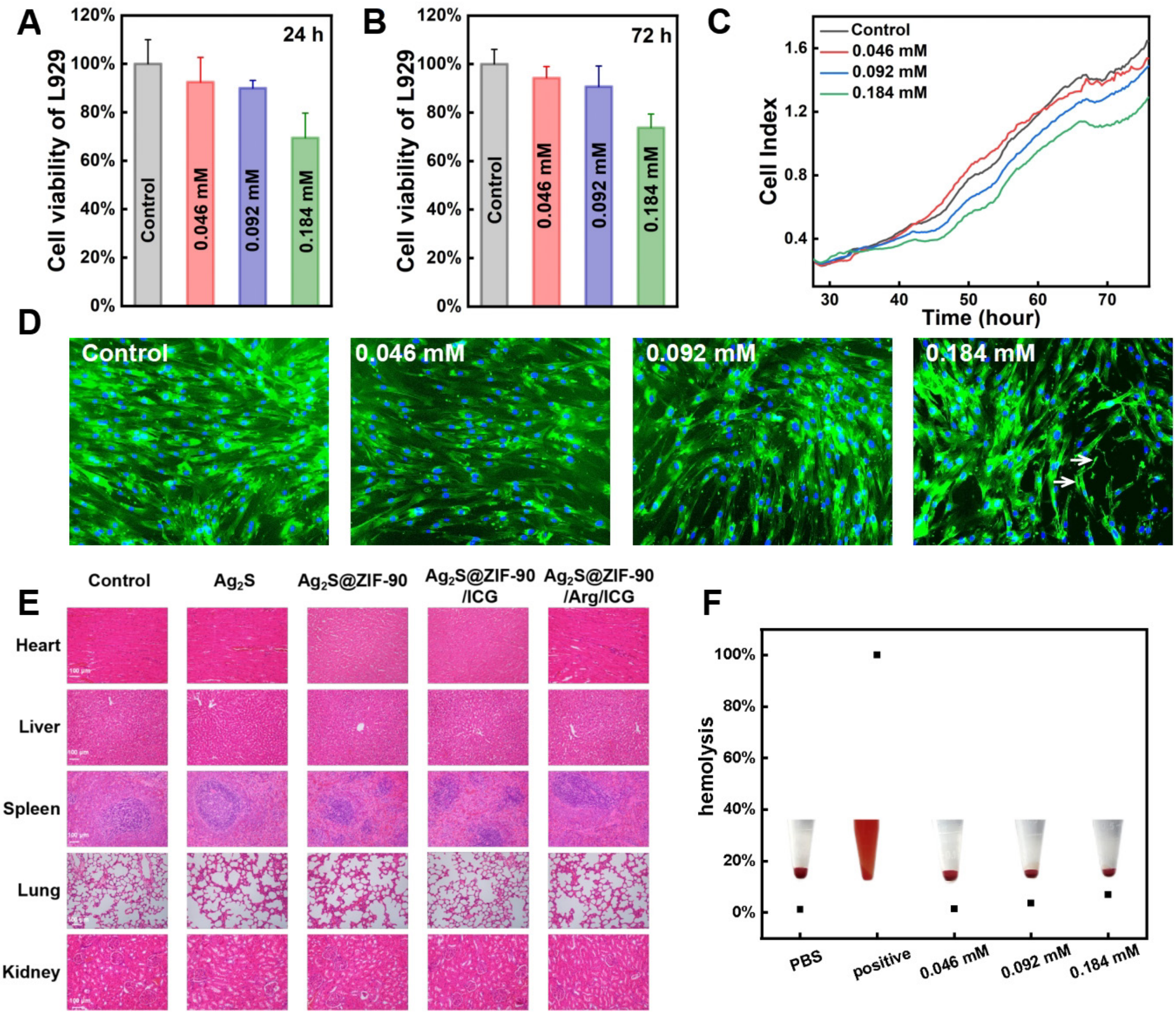

Figure S29. **Dark toxicity of Ag_2S@ZIF-90/Arg/ICG NCs.** (A) and (B) The cell viability of L929 mouse fibroblast cells 24 h and 72 h with containing 0 mM, 0.046 mM, 0.092 mM, 0.184 mM of Ag_2S@ZIF-90/Arg/ICG NCs. (C) The proliferation of L929 mouse fibroblast cells with different concentration Ag_2S@ZIF-90/Arg/ICG NCs by RTCA. (D) CLSM images of HGFs incubated with Ag_2S@ZIF-90/Arg/ICG NCs (0 mM, 0.046 mM, 0.092 mM, 0.184 mM). Arrows: apoptotic cells. (E)H&E staining of major organs (including heart, liver, lung, spleen, and kidney) after various treatments. Scale bar, 100 μm. (F) Photographs and analysis of hemolysis of human RBCs in the presence of different concentration (including 0 mM, 0.046 mM, 0.092 mM, 0.184 mM) of Ag_2S@ZIF-90/Arg/ICG NCs: Triton-X 100 and PBS were used as positive control and negative control, respectively.

References

1. Lv L, Wang H. Ag_2S nanorice: hydrothermal synthesis and characterization study. Mater Lett. 2014; 121: 105-8.
2. Nosike EI, Jiang Z, Miao L, Akakuru OU, Yuan B, Wu S, et al. A novel hybrid nanoadsorbent for effective Hg^{2+} adsorption based on zeolitic imidazolate framework (ZIF-90) assembled onto poly acrylic acid capped Fe_3O_4 nanoparticles and cysteine. J Hazard Mater. 2020; 392: 122288.
3. Yuan Z, Lin C, He Y, Tao B, Chen M, Zhang J, et al. Near-infrared light-triggered nitric-oxide-enhanced photodynamic therapy and low-temperature photothermal therapy for biofilm elimination. ACS Nano. 2020; 14: 3546-62.
4. Qi M, Ren X, Li W, Sun Y, Sun X, Li C, et al. NIR responsive nitric oxide nanogenerator for enhanced biofilm eradication and inflammation immunotherapy against periodontal diseases. Nano Today. 2022; 43: 101447.
5. Montesinos VN, Sleiman M, Cohn S, Litter MI, Destaillats H. Detection and quantification of reactive oxygen species (ROS) in indoor air. Talanta. 2015; 138: 20-7.
6. Li Z, Li Z, Holamoge YV, Zaid W, Osborn ML, Bhatta S, et al. Mouthwash to deliver indocyanine green for near infrared dental fluorescence imaging. IEEE J Quantum Electron. 2020; 27: 1-8.
7. Sun Y, Sun X, Li X, Li W, Li C, Zhou Y, et al. A versatile nanocomposite based on nanoceria for antibacterial enhancement and protection from aPDT-aggravated inflammation via modulation of macrophage polarization. Biomaterials. 2021; 268: 120614.
